# Comparison of neonatal outcomes of small for gestational age and appropriate for gestational age preterm infants born at 28–36 weeks of gestation: a multicentre study in Ethiopia

**DOI:** 10.1136/bmjpo-2020-000740

**Published:** 2020-09-15

**Authors:** Netsanet Workneh Gidi, Robert L Goldenberg, Assaye K Nigussie, Elizabeth McClure, Amha Mekasha, Bogale Worku, Matthias Siebeck, Orsolya Genzel-Boroviczeny, Lulu M Muhe

**Affiliations:** 1 Pediatric and Child Health, Jimma University, Jimma, Oromia, Ethiopia; 2 Center for International Health, University Hospital, LMU, Munich, Germany; 3 Department of Obstetrics and Gynecology, Columbia University, New York, New York, USA; 4 Newborn & Child Health, Bill and Melinda Gates Foundation, Seattle, Washington, USA; 5 Center for Clinical Research Network Coordination, RTI International, Durham, North Carolina, USA; 6 Pediatrics and Child Heath, Addis Ababa University College of Health Sciences, Addis Ababa, Oromia, Ethiopia; 7 Ethiopian Pediatric Society, Addis Ababa, Ethiopia; 8 Institute for Medical Education, University Hospital, LMU, Munich, Germany; 9 Dr. von Hauner University Children’s Hospital, University Hospital, LMU, Munich, Germany

**Keywords:** neonatology, mortality

## Abstract

**Purpose:**

The aim of this study was to assess morbidity and mortality pattern of small for gestational age (SGA) preterm infants in comparison to appropriate for gestational age (AGA) preterm infants of similar gestational age.

**Method:**

We compared neonatal outcomes of 1336, 1:1 matched, singleton SGA and AGA preterm infants based on their gestational age using data from the study ‘Causes of Illness and Death of Preterm Infants in Ethiopia (SIP)’. Data were analysed using SPSS V.23. ORs and 95% CIs and χ^2^ tests were done, p value of <0.05 was considered statistically significant.

**Result:**

The majority of the infants (1194, 89%) were moderate to late preterm (32–36 weeks of gestation), 763 (57%) were females. Male preterm infants had higher risk of being SGA than female infants (p<0.001). SGA infants had increased risk of hypoglycaemic (OR and 95% CI 1.6 (1.2 to 2.0), necrotising enterocolitis (NEC) 2.3 (1.2 to 4.1), polycythaemia 3.0 (1.6 to 5.4), late-onset neonatal sepsis (LOS) 3.6 (1.1 to 10.9)) and prolonged hospitalisation 2.9 (2.0 to 4.2). The rates of respiratory distress syndrome (RDS), apnoea and mortality were similar in the SGA and AGA groups.

**Conclusion:**

Neonatal complications such as hypoglycaemic, NEC, LOS, polycythaemia and prolonged hospitalisation are more common in SGA infants, while rates of RDS and mortality are similar in SGA and AGA groups. Early recognition of SGA status, high index of suspicion and screening for complications associated and timely intervention to prevent complications need due consideration.

**What is known about the subject?**

- Intrauterine growth restriction is one of the common perinatal complications associated with increased neonatal morbidity and mortality.
- As an adaptation to early extrauterine life, in utero stress associated with placental insufficiency increases secretion of steroid hormones in the fetus.
- This results in acceleration of brain and lung maturation, however several studies have reported contradictory findings on the morbidity and mortality patterns of small for gestational age (SGA) infants.

**What this study adds?**

- Accelerated maturity associated with intrauterine growth restriction expected in SGA preterm infants did not protect them from RDS and mortality.
- Rather, SGA infants have significantly increased risk of hypoglycaemic, necrotising enterocolitis, polycythaemia, late-onset neonatal sepsis and prolonged hospitalisation.

## Introduction

Globally, intrauterine growth restriction (IUGR) occurs in about 24% of newborns per year, and the majority are born in low-income and middle-income countries (LMICs).^1^ IUGR is one of the rising public health challenges, because it contributes to increased risk of neonatal morbidity and mortality, and chronic diseases in adulthood.^2–4^ Small for gestational age (SGA) is commonly defined as birthweight-for-gestational-age measure below the 10th percentile compared with a gender-specific reference population.^5^ IUGR refers to a condition in which the fetal growth is slower than normal, a common cause of SGA; while SGA includes constitutionally small babies.^2^ IUGR and SGA can only be distinguished if serial prenatal ultrasound evaluations are done. IUGR occurs due to compromised fetal growth usually related to placental malfunction for various reasons, such as maternal hypertension, diabetes, cardiopulmonary disease, anaemia, malnutrition and multiple pregnancies.^6 7^ Congenital malformations and fetal infection may also lead to SGA. IUGR and SGA are commonly used interchangeably and for this paper we will refer to these conditions as SGA.^6^

Conditions, such as multiple pregnancies or pregnancies complicated by maternal hypertension or other placental dysfunction, cause increased secretion of glucocorticoids, other steroid hormones and catecholamines that results in acceleration of brain and lung maturation by as much as 3–4 weeks or more when compared with the appropriate for gestational age (AGA) infants of the same gestational age (GA); this is considered an adaptation to early extrauterine life.^8–10^ However, this adaptive response may fail if the placental dysfunction progresses and the fetus experiences severe anoxia and malnutrition that could result in increased risk of complications and death.^2 10^

Several studies have reported contradictory findings on the effect IUGR on neonatal respiratory distress syndrome (RDS).^11^ The risk of RDS has been reported to be same or lower in SGA infants compared with AGA infants of similar GA,^12–14^ but numerous studies have reported increased risk of morbidity and mortality in SGA infants.^3 13 15–19^

The reported conflicting findings of neonatal outcomes of SGA infants could be due to the differences in the timing of the onset of placental insufficiency, the severity of growth restriction and the degree of cardiovascular adaptation.^20^ Differences in settings of the studies could play a role in terms of early diagnoses of high-risk pregnancies and timely intervention, which could abort the progression of the insult and prevent complications.

SGA infants are at higher risk of metabolic and haematological disturbances and those with severe SGA are more likely to die during the neonatal period.^10 18^ Those who survive the neonatal period have a high risk of growth and developmental impairment in childhood, and metabolic, hormonal and cognitive disorders later in adulthood.^7 21^ Reports from high-income countries show no significant mortality difference between preterm SGA and AGA infants. However, in LMICs preterm SGA infants have increased risk of mortality.^6^ Most of the studies on preterm infants’ health and SGA are reported from high-income countries and there is a paucity of data from LMICs where the burden is very high.^22^ The aim of this study is to assess morbidity and mortality pattern of preterm SGA infants in comparison to AGA infants of similar GA in five neonatal intensive care units (NICUs) in Ethiopia.

## Method

We analysed maternal obstetric and clinical data of GA-matched SGA and AGA preterm infants admitted to NICUs from a study on Causes of Illness and Death of Preterm Infants in Ethiopia (SIP). SIP was a prospective descriptive multisite hospital-based study conducted in five selected hospitals in Ethiopia. The protocol and the primary result have been published.^23 24^

After exclusion of multiple births, those with congenital malformations and chromosomal disorders and large for gestational age infants, SGA infants were identified and 1:1 match with AGA preterm infants was done randomly. Weight for GA was assessed based on gender-specific and GA-specific Fenton growth charts.^25^ SGA was defined as a birth weight below the 10th percentile for GA, and AGA birth weight was defined as between the 10th and 90th percentile for GA. The association of SGA with gender, mortality, length of hospital stay, clinical diagnoses such as RDS, necrotising enterocolitis (NEC), neonatal infections, hypoglycaemic, perinatal asphyxia and polycythaemia were analysed. Maternal obstetric variables such as maternal age, marital status, pregnancy-induced hypertension, premature rupture of membranes (PROM), antepartum haemorrhage, chorioamnionitis, dexamethasone administration and mode of delivery were assessed for association with birth weight for GA.

GA estimation was based on maternal menstrual history, early fetal ultrasound or New Ballard Score examination. Complications of preterm birth such as RDS and neonatal infections were diagnosed based on clinical findings and investigations including chest X-ray, blood culture and white blood cell count. Death before the 28th day of life was defined as neonatal mortality. Statistical analysis was done using the SPSS V.23 statistical program. Differences in association of the variables were analysed with χ^2^ tests and a p value of <0.05 was considered significant. ORs and 95% CIs were calculated to identify clinical variables associated with SGA.

### Patient and public involvement statement

Patients were not involved in the design, recruitment and conduct of this study.

## Result

**Figure 1:**
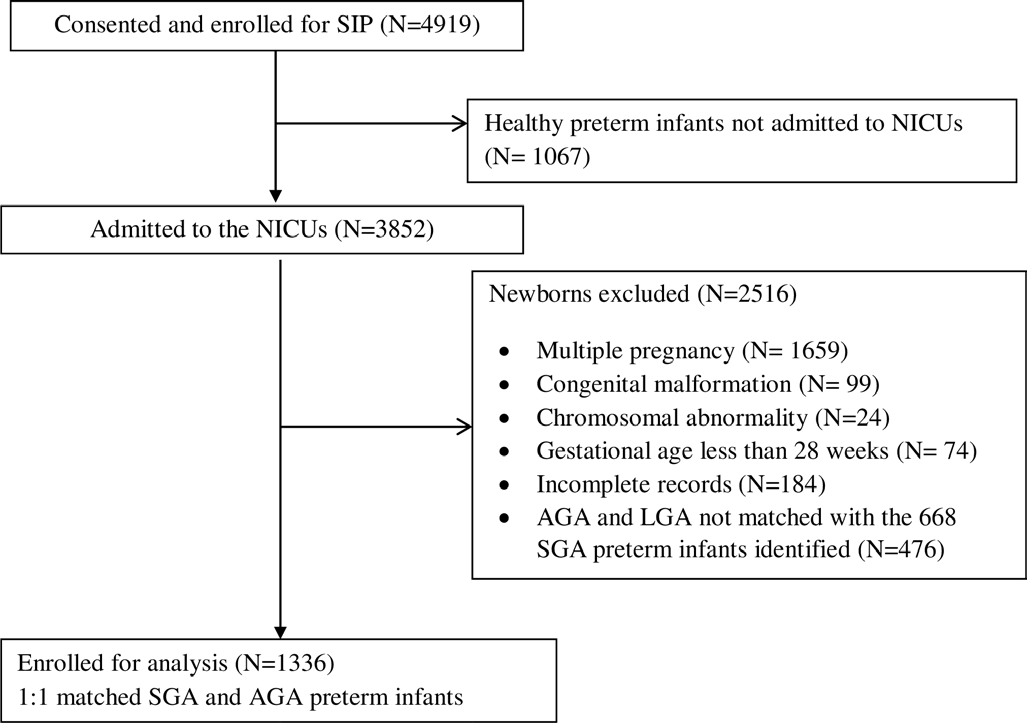
A total of 1336 singleton SGA and AGA preterm infants were eligible for the study. AGA, appropriate for gestational age; NICU, neonatal intensive care unit; SGA, small for gestational age; SIP, Causes of Illness and Death of Preterm Infants in Ethiopia.

A total of 1336 singleton SGA and AGA preterm infants were eligible for the study (figure 1); 763 (57%) were females. The majority of the infants, 1094 (81.9%) were moderate to late preterm (32–36 weeks of GA), and 893 (67%) of the infants had a birth weight >1500 g. The common complications the infants had are shown in table 1.

**Table 1:** Preterm infants’ perinatal data

Variables	No (%)
Infants’ sex	
Male	573 (42.9)
Female	763 (57.1)
Birth weight (g)	
<1000	78 (5.8)
1000–1499	365 (27.3)
1500–1999	538 (40.3)
≥2000	355 (26.6)
Gestational age (weeks)	
28–32	242 (18.1)
33–34	562 (42.1)
35–36	532 (39.8)
Common morbidities	
Neonatal infections	696 (52.1)
Respiratory distress syndrome	514 (38.5)
Hypoglycaemic	326 (24.4)
Hyperbilirubinaemia	417 (31.2)
Perinatal asphyxia	98 (7.3)
Polycythaemia	58 (4.3)
Distribution of study subjects by hospitals	
Gondar University Hospital	373 (27.9)
Saint Paul Millennium College Hospital	358 (26.8)
Black Lion Hospital	307 (23.0)
Ghandi Memorial Hospital	158 (11.8)
Jimma University Medical Center	140 (10.5)

The male preterm infants had a higher risk of SGA than female infants (p<0.001). Maternal age and marital status were not associated with SGA, while pregnancy induced hypertension had a statistically significant association with SGA. SGA infants were more likely to be delivered by caesarian section than AGA preterm infants (p<0.001). Prophylactic dexamethasone was given more often to the mothers of the preterm infants who were SGA (p=0.017). Other obstetric factors studied such as PROM, antepartum haemorrhage and chorioamnionitis were more common in mothers of AGA preterm infants (p<0.01) (table 2).

**Table 2:** Factors associated with birth weight for gestational age

Variables, no (%)	Total	SGA N=668	AGA N=668	P value
Maternal age (years)				0.303
<20	236 (17.7)	115 (17.2)	121 (18.1)	
20–34	980 (73.4)	485 (72.6)	495 (74.1)	
≥35	120 (9.0)	88 (13.2)	52 (7.9)	
Marital status				0.207
Married	1283 (96.0)	637 (95.4)	647 (96.9)	
Single	53 (4.0)	31 (4.6)	21 (3.1)	
Mode of delivery				<0.001
Caesarean section	509 (38.1)	283 (42.3)	226 (33.3)	
Vaginal delivery	827 (61.9)	385 (57.3)	442 (66.1)	
Major obstetric complications				
Pregnancy-induced hypertension	445 (33.3)	290 (43.4)	155 (23.2)	<0.001
PROM	191 (14.4)	72 (10.8)	120 (17.9)	<0.001
Antepartum haemorrhage	158 (11.8)	63 (9.4)	95 (14.2)	0.007
Chorioamnionitis	61 (4.6)	14 (2.1)	47 (7.0)	<0.001
Mother received dexamethasone	435 (32.6)	238 (35.6)	197 (29.5)	0.017
Sex of the infant				<0.001
Male	573 (42.9)	353 (52.8)	220 (32.9)	
Female	763 (57.1)	315 (47.2)	448 (67.1)	

AGA, appropriate for gestational age; PROM, premature rupture of membranes; SGA, small for gestational age.

The rates of RDS, apnoea and mortality were similar among the SGA and AGA groups. While SGA infants had a 1.6 times higher risk of developing hypoglycaemic, p<0.001, OR 1.58, 95% CI 1.23 to 2.04. NEC was diagnosed in 5.2% of SGA and 2.4% of AGA infants, p=0.007, OR 2.25, 95% CI 1.24 to 4.11. Polycythaemia was seen more often in SGA infants, p<0.001, OR 3.00, 95% CI 1.65 to 5.45. Similar rates of neonatal infections were seen in both groups, while SGA infants had 3.6 times higher risk of developing late-onset neonatal sepsis than AGA preterm infants, p=0.018, OR 3.55 95% CI 1.16 to 10.85. SGA infants were more likely to be hospitalised for >21 days than AGA preterm infants, p<0.001, OR 2.90, 95% CI 1.98 to 4.24 (table 3).

**Table 3:** Comparison of neonatal outcomes of small for gestational age and appropriate for gestational age preterm infants

Variables	SGA (N=668)	AGA (N=668)	P value	OR	95% CI	
					Lower	Upper
RDS	257 (38.5)	257 (38.5)	1.00	1.00	0.80	1.25
Apnoea	66 (9.9)	54 (8.0)	0.251	1.25	0.86	1.82
Hypoglycaemic	191 (28.6)	135 (20.2)	<0.001	1.58	1.23	2.04
NEC	35 (5.2)	16 (2.4)	0.007	2.25	1.24	4.11
Polycythaemia	43 (6.4)	15 (2.2)	<0.001	3.00	1.65	5.45
EOS	275 (41.2)	271 (40.6)	0.824	1.03	0.82	1.28
LOS	14 (2.1)	4 (0.6)	0.018	3.55	1.16	10.85
Hyperbilirubinaemia	196	221	0.140	0.84	0.67	1.06
Perinatal asphyxia	48	50	0.834	0.96	0.63	1.44
Mortality	51 (7.6)	51 (7.6)	1.00	1.00	0.67	1.50
Length of hospital stay						
<21 days	564 (84.4)	628 (94.0)	–	–	–	–
≥21 days	104 (15.6)	40 (6.0)	<0.001	2.90	2.98	4.24

AGA, appropriate for gestational age; EOS, early onset neonatal sepsis; LOS, late-onset neonatal sepsis; NEC, necrotising enterocolitis; RDS, respiratory distress syndrome; SGA, small for gestational age.

## Discussion

Comparison of neonatal outcomes of SGA and AGA preterm infants remains controversial, as several investigators have reported conflicting results. In the current study, the rates of RDS and mortality among the two groups were similar, unlike the 2 to 4 times,^6^ and 16 times increased mortality of SGA preterm infants reported from LMICs.^22^ The mortality rate in this study could have been partly modified related to the antenatal dexamethasone the SGA groups had received more than the AGA infants. These findings were in line with the report of Bartal *et al* among late preterm neonates.^14^ And our findings contradict the reports of Tsai *et al* and Tayson *et al*, who reported an increased risk of RDS and mortality among SGA infants.^13 26^ However, Bartels *et al* and *Sharma et al* reported increased risk of death and decreased risk of RDS.^12 19^ The contradicting findings in the literature might be due to the multifactorial nature of outcome of SGA, the cause of SGA and severity of the condition, duration of intrauterine hypoxia and variations in settings of the studies. The GA at birth could modify the physiological changes and adaptation to extrauterine environment.

The SGA infants were more likely to be delivered by caesarean section (p<0.001) and their mothers were given prophylactic dexamethasone more often compared with mothers of AGA infants (p=0.017), this may have improved the overall outcome of the SGA infants. Pregnancy-induced hypertension was associated with the SGA (p<0.001), whereas acute obstetric condition such as antepartum haemorrhage, chorioamnionitis and PROM were more common in mothers of AGA infants. This finding is similar to the report of Boghossian *et al*.^27^

The rate of early onset neonatal infection was comparable in both groups, however SGA infants had a higher risk of late-onset neonatal sepsis. Similarly, SGA infants had an increased risk of NEC and hypoglycaemic compared with AGA infants. These findings are consistent with reports of Hasthi *et al* from India and Boghossian *et al* from USA.^15 27^ These can likely be explained by the severe undernutrition the infants experienced predisposing them to infection, although the mechanisms of how undernutrition is related to immune suppression is not well understood, there are strong epidemiological data supporting the link.^28^ The increased risk of NEC might be associated with immature gut development that has resulted from intrauterine chronic fetal hypoxia and consequent cardiovascular redistribution of blood flow away from the gastrointestinal tract to vital organs.^29^

SGA infants had a statistically significant increased risk of prolonged hospitalisation for >21 days, likely related to the severity of the morbidities they had; Sharma *et al* from the USA reported a similar finding in a retrospective study involving 2530 infants born at ≤36 weeks.^19^ Polycythaemia (a venous haematocrit >65%) can occur as a response to intrauterine hypoxia, the hyperviscosity of blood associated might result in serious complications.^30^ SGA infants are at higher risk of developing polycythaemia.^2^ Similarly, we found a threefold increased risk of polycythaemia in SGA compared with AGA infants. The observed complications could be prevented with improvement of neonatal care.

## Conclusion

The SGA infants in this study had increased risk of hypoglycaemic, NEC, late-onset neonatal sepsis, polycythaemia and prolonged hospitalisation. The rates of RDS and neonatal mortality were similar in SGA and AGA infants. Proper antenatal care, timely recognition of high-risk pregnancies and right interventions are needed to prevent IUGR and the subsequent SGA-related complications. Screening for morbidities associated with SGA, preventive measures and adequate postnatal care could contribute for improvement of neonatal outcomes.

## Supplementary Material

Author's manuscript
