# Supplementary material for: Comparison of neonatal outcomes of small for gestational age and appropriate for gestational age preterm infants born at 28–36 weeks of gestation: a multicentre study in Ethiopia
Source: BMJ Paediatr Open. 2020 Sep 15;4(1):e000740. doi: 10.1136/bmjpo-2020-000740 (PMC7493091; doi:10.1136/bmjpo-2020-000740)
Supplement: Author's manuscript [file bmjpo-2020-000740.draft_revisions.pdf]

BMJ Paediatrics Open

BMJ Paediatrics Open is committed to open peer review. As part of this commitment we make the peer review history of every article we publish publicly available.

When an article is published we post the peer reviewers' comments and the authors' responses online. We also post the versions of the paper that were used during peer review. These are the versions that the peer review comments apply to.

The versions of the paper that follow are the versions that were submitted during the peer review process. They are not the versions of record or the final published versions. They should not be cited or distributed as the published version of this manuscript.

BMJ Paediatrics Open is an open access journal and the full, final, typeset and author-corrected version of record of the manuscript is available on our site with no access controls, subscription charges or pay-per-view fees (http://bmjpaedsopen.bmj.com).

If you have any questions on BMJ Paediatrics Open's open peer review process please email info.bmjpo@bmj.com

BMJ Paediatrics Open

Comparison of neonatal outcomes of small for gestational age and appropriate for gestational age preterm infants born at 28 to 36 weeks of gestation

Journal:	*BMJ Paediatrics Open*
Manuscript ID	bmjpo-2020-000740
Article Type:	Original research
Date Submitted by the Author:	21-May-2020

Complete List of Authors:	Gidi, Netsanet; Jimma University College of Public Health and Medical Sciences, Pediatric and child health; Ludwig-Maximilians-Universitat Munchen Center for International Health<br>Goldenberg , Robert L. ; Columbia University<br>Nigussie, Assaye K. ; Bill and Melinda Gates Foundation<br>McClure, Elizabeth; RTI International<br>Mekasha, Amha; Addis Ababa University College of Health Sciences<br>Worku, Bogale; Ethiopian Pediatric Society<br>Siebeck, Matthias; Medical Center of the University of Munich (LMU)<br>Genzel-Boroviczeny, Orsolya; Ludwig Maximilian University of Munich , Dr. von Hauner University Children's Hospital<br>Muhe, Lulu M; Addis Ababa University College of Health Sciences

Keywords:	Neonatology, Mortality

SCHOLARONE™
Manuscripts

I, the Submitting Author has the right to grant and does grant on behalf of all authors of the Work (as defined in the below author licence), an exclusive licence and/or a non-exclusive licence for contributions from authors who are: i) UK Crown employees; ii) where BMJ has agreed a CC-BY licence shall apply, and/or iii) in accordance with the terms applicable for US Federal Government officers or employees acting as part of their official duties; on a worldwide, perpetual, irrevocable, royalty-free basis to BMJ Publishing Group Ltd ("BMJ") its licensees and where the relevant Journal is co-owned by BMJ to the co-owners of the Journal, to publish the Work in this journal and any other BMJ products and to exploit all rights, as set out in our licence.

The Submitting Author accepts and understands that any supply made under these terms is made by BMJ to the Submitting Author unless you are acting as an employee on behalf of your employer or a postgraduate student of an affiliated institution which is paying any applicable article publishing charge ("APC") for Open Access articles. Where the Submitting Author wishes to make the Work available on an Open Access basis (and intends to pay the relevant APC), the terms of reuse of such Open Access shall be governed by a Creative Commons licence – details of these licences and which Creative Commons licence will apply to this Work are set out in our licence referred to above.

Other than as permitted in any relevant BMJ Author's Self Archiving Policies, I confirm this Work has not been accepted for publication elsewhere, is not being considered for publication elsewhere and does not duplicate material already published. I confirm all authors consent to publication of this Work and authorise the granting of this licence.

Confidential: For Review Only

Title: Comparison of neonatal outcomes of small for gestational age and appropriate for gestational age preterm infants born at 28 to 36 weeks of gestation

Authors: Netsanet Workneh Gidi[a,h], Robert L. Goldenberg[b], Assaye K. Nigussie[c], Elizabeth M. McClure[d], Amha Mekasha[e], Bogale Worku[f], Matthias Siebeck[g], Orsolya Genzel-Boroviczény[g], Lulu M. Muhe[e]

Affiliations

[a] Institute of Health Sciences, Jimma University, Jimma, Ethiopia

[b] Columbia University, New York, NY, USA

[c] Bill and Melinda Gates Foundation, Seattle, WA, USA

[d] RTI International, 3040 Cornwallis Rd., Durham, NC, 27709, USA

[e] College of Health Sciences, Addis Ababa University, Addis Ababa, Ethiopia

[f] Ethiopian Pediatric Society, Addis Ababa, Ethiopia

[g] LMU University Hospital, Munich, Germany

[h] Center for International Health, LMU University Hospital, Munich, Germany

Corresponding author: Netsanet Workneh (MD, DTM&H)

College of public health and medical sciences, Jimma University, Jimma, Ethiopia

PhD candidate, at CIHLMU, Munich, Germany

Phone: +251 917762109

Email: konetsanet@gmail.com, or Netsanet.Workneh@lrz.uni-muenchen.de

Word count: 1912

For peer review only

ABSTRACT

Purpose: The aim of this study was to assess morbidity and mortality pattern of small for gestational age (SGA) preterm infants in comparison to appropriate for gestational age (AGA) preterm infants of similar gestational age.

Method: We compared neonatal outcomes of 1336, 1:1 matched, singleton SGA and AGA preterm infants based on their gestational age using data from the study "Causes of Illness and Death of Preterm Infants in Ethiopia (SIP)". Data were analyzed using SPSS version 23. Chi square test, odds ratio and 95 % confidence intervals (95%CI) were done, p-value of <0.05 was considered statistically significant.

Result: The majority of the infants 1194 (89%) were moderate to late preterm (32 to 36 weeks of gestation), 763 (57%) were females. Male preterm infants had higher risk of being SGA than female infants, 61.6% versus 41.3%. SGA infants had increased risk of hypoglycemia (OR and 95% CI of 1.6 (1.2-2.0), necrotizing enterocolitis (NEC) 2.3(1.2-4.1), polycythemia 3.0 (1.6-5.4), late onset neonatal sepsis (LOS) 3.6 (1.1-10.9)) and prolonged hospitalization 2.9 (2.0-4.2). The rates of respiratory distress syndrome (RDS), apnea and mortality were similar in the SGA and AGA groups.

Conclusion: Neonatal complications such as hypoglycemia, NEC, LOS, polycythemia and prolonged hospitalization are more common in SGA infants; while rates of RDS and mortality are similar in SGA and AGA groups. Early recognition of SGA status, high index of suspicion and screening for complications associated, and timely intervention to prevent complications need due consideration.

For Review Only

INTRODUCTION

Globally, intrauterine growth restriction (IUGR) occurs in about 24% of newborns per year, and the majority are born in developing countries.[1] IUGR is one of the rising public health challenges, because it contributes to increased risk of neonatal morbidity and mortality, and chronic diseases in adulthood.[2-4] Small for gestational age (SGA) is commonly defined as birthweight-for-gestational-age measure below the 10th percentile compared to a gender-specific reference population.[5] IUGR and SGA can only be distinguished if prenatal ultra sounds are available. IUGR occurs due to compromised fetal growth usually due to placental malfunction for various reasons, such as maternal hypertension, diabetes, cardiopulmonary disease, anemia, malnutrition and multiple pregnancies.[6, 7] Congenital malformations and fetal infection may also lead to SGA. IUGR and SGA are commonly used interchangeably and for this paper we will refer to these conditions as SGA.[6]

Conditions, such as multiple pregnancies or pregnancies complicated by maternal hypertension or other placental dysfunction, cause increased secretion of glucocorticoids, other steroid hormones and catecholamines that results in acceleration of brain and lung maturation by as much as 3 to 4 weeks or more when compared to the AGA infants of the same gestational age (GA); this is considered an adaptation to early extrauterine life.[9-11] However, this adaptive response may fail if the placental dysfunction progresses and the fetus experiences severe anoxia and malnutrition that could result in increased risk of complications and death.[2, 11]

Several studies have reported contradictory findings on the morbidity and mortality patterns of SGA infants.[8] The risk of respiratory distress syndrome (RDS) has been reported to be same or lower in SGA infants compared to AGA infants of similar GA,[12-14] but numerous studies have reported increased risk of morbidity and mortality in SGA infants.[3,13,15-19] Neonatal outcomes of SGA infants generally depend on the timing of the onset of placental insufficiency, the severity of growth restriction, the degree of cardiovascular adaptation, and GA at birth.[20]

SGA infants are at higher risk of metabolic and hematological disturbances and those with severe SGA are more likely to die during the neonatal period.[11, 18] Those who survive the neonatal period have a high risk of growth and developmental impairment in childhood, and metabolic, hormonal and cognitive disorders later in adulthood.[7, 21] Most of the studies on preterm infants' health and SGA are reported from developed countries and there is a paucity of data from low- income countries where the burden is very high.[22] The aim of this study is to assess morbidity and mortality pattern of SGA infants in comparison to AGA infants of same GA.

METHOD

Data source

We analyzed maternal obstetric and clinical data of GA matched SGA and AGA preterm infants admitted to neonatal intensive units (NICUs) from a study on Causes of Illness and Death of Preterm Infants in Ethiopia (SIP). SIP was a prospective descriptive multisite hospital-based study conducted in 5 selected hospitals in Ethiopia. The protocol and the primary result have been published.[23, 24]

Patient involvement

Patients were not directly involved in design, patient recruitment or plan to disseminate the study results.

Inclusion and exclusion criteria

After exclusion of multiple births, those with congenital malformations and chromosomal disorders and large for gestational age (LGA) infants, SGA infants were identified and 1:1 match with AGA preterm infants was done randomly. Weight for GA was assessed based on gender and GA specific Fenton growth charts.[25] SGA was defined as a birth weight below the 10th percentile for GA, and AGA birth weight was defined as between the 10th and 90th percentile for GA.

Outcome variables

The association of SGA with gender, mortality, length of hospital stay, clinical diagnoses such as RDS, necrotizing enterocolitis (NEC), neonatal infections, hypoglycemia, perinatal asphyxia and polycythemia were analyzed. Maternal obstetric variables such as maternal age, marital status, pregnancy induced hypertension, premature rupture of membranes (PROM), antepartum hemorrhage, chorioamnionitis, dexamethasone administration and mode of delivery were assessed for association with birth weight for GA.

GA estimation was based on maternal menstrual history, early fetal ultrasound or New Ballard Score examination. Complications of preterm birth such as RDS and neonatal infections were diagnosed based on clinical findings and investigations including chest x-ray, blood culture and white blood cell count. Death before the 28th day of life was defined as neonatal mortality.

Statistical analysis

Statistical Analysis was done using the SPSS statistical program. Differences in association of the outcome variables were analyzed with Chi-square tests and a p-value of <0.05 was considered significant. Odds ratios (OR) and 95% confidence intervals (95% CI) were calculated to identify clinical variables associated with SGA.

Ethical approval was obtained from Addis Ababa University College of health Sciences institutional review board (Ethics ID: AAUMF 03-008) before the study was commenced. The parents of the infants were given adequate information and asked for informed consent prior to participation.

RESULT

A total of 1336 singleton SGA and AGA preterm infants were eligible for the study (Figure1); 763 (57%) were females. The majority of the infants, 1194 (89%) were moderate to late preterm (32 to 36 weeks of GA), and 893 (67%) of the infants had a birthweight greater than 1500 grams. The common complications the infants had are shown in table 1.

Confidential: For Review Only

Table 1. Preterm infants' perinatal data

**Variables**	**No (%)**
**Infants sex**	
Male	573 (42.9)
Female	763 (57.1)
**Birthweight in grams**	
<1000	78 (5.8)
1000 to 1499	365 (27.3)
1500 to 1999	538 (40.3)
≥2000	355 (26.6)
**Gestational Age in weeks**	
28 to 32	242 (18.1)
33 to 34	562 (42.1)
35 to 36	532 (38.8)
**Common morbidities**	
Neonatal infections	696 (52.1)
Respiratory distress syndrome	514 (38.5)
Hypoglycemia	326 (24.4)
Hyperbilirubinemia	417 (31.2)
Perinatal asphyxia	98 (7.3)
Polycythemia	58 (4.3)

The male preterm infants had a higher risk of SGA than female infants of similar GA, 61.6% versus 41.3%, (p-value =.000). Maternal age and marital status were not associated with SGA, while pregnancy induced hypertension had a statistically significant association with SGA. SGA infants were more likely to be delivered by caesarian section than AGA preterm infants, (p-value=.000). Prophylactic dexamethasone was given more often to the mothers of the preterm infants who were SGA, (p-value = .017). Other obstetric factors studied such as PROM, antepartum hemorrhage, and chorioamnionitis were more common in mothers of AGA preterm infants, (p-value <0.01) (Table 2).

Confidential: For Review Only

Table 2. Factors associated with birth weight for gestational age.

Variables, No (%)	Total	SGA N=668	AGA N=668	P-value
**Maternal age**				.303
<20 years	236 (17.7)	115 (17.2)	121 (18.1)	
20 to 34 years	980 (73.4)	485 (72.6)	495 (74.1)	
≥35 years	120 (9.0)	88 (13.2)	52 (7.9)	
**Marital Status**				.207
Married	1283 (96.0)	637 (95.4)	647 (96.9)	
Single	53 (4.0)	31(4.6)	21 (3.1)	
**Mode of Delivery**				.000
Cesarean section	509 (38.1)	283 (42.3)	226 (33.3)	
Vaginal delivery	827 (61.9)	385 (57.3)	442 (66.1)	
**Major obstetric complications**				
Pregnancy induced hypertension	445 (33.3)	290 (43.4)	155 (23.2)	.000
PROM	191 (14.4)	72 (10.8)	120 (17.9)	.000
Antepartum hemorrhage	158 (11.8)	63 (9.4)	95 (14.2)	.007
Chorioamnionitis	61 (4.6)	14 (2.1)	47 (7.0)	.000
Mother received dexamethasone	435 (32.6)	238 (35.6)	197 (29.5)	.017
**Sex of the infant**				.000
Male	573 (42.9)	353 (52.8)	220 (32.9)	
Female	763 (57.1)	315 (47.2)	448 (67.1)	

AGA= Appropriate for gestational age, SGA = Small for gestational age, PROM= Premature rupture of membranes

The rates of RDS, apnea and mortality were similar among the SGA and AGA groups. While SGA infants had a 1.6 times higher risk of developing hypoglycemia, p-value = .000, OR = 1.58, 95% CI (1.23-2.04). NEC was diagnosed in 5.2% of SGA and 2.4% of AGA infants, p-value =.007, odds ratio = 2.25 95% CI (1.24-4.11). Polycythemia was seen more often in SGA infants,

Confidential: For Review Only

p- value =.000, OR=3.00 95% CI (1.65-5.45). Similar rates of neonatal infections were seen in both groups, while SGA infants had 3.6 times higher risk of developing late onset neonatal sepsis than AGA preterm infants, p-value=.018, OR = 3.55 95%CI (1.16-10.85). SGA infants were more likely to be hospitalized for more than 21 days than AGA preterm infants, p-value =.000, OR=2.90, 95%CI (1.98-4.24) (Table 3).

Table 3. Comparison of neonatal outcomes of small for gestational age and appropriate for gestational age preterm infants

Variables	SGA (N=668)	AGA (N=668)	P-value	Odds ratio	95 % CI	
					Lower	Upper
RDS	257 (38.5)	257 (38.5)	1.00	1.00	.80	1.25
Apnea	66 (9.9)	54 (8.0)	.251	1.25	.86	1.82
Hypoglycemia	191 (28.6)	135 (20.2)	.000	1.58	1.23	2.04
NEC	35 (5.2)	16 (2.4)	.007	2.25	1.24	4.11
Polycythemia	43 (6.4)	15 (2.2)	.000	3.00	1.65	5.45
EOS	275 (41.2)	271 (40.6)	.824	1.03	.82	1.28
LOS	14 (2.1)	4 (0.6)	.018	3.55	1.16	10.85
Hyperbilirubinemia	196	221	.140	.84	.67	1.06
Perinatal asphyxia	48	50	.834	.96	.63	1.44
Mortality	51 (7.6)	51 (7.6)	1.00	1.00	.67	1.50
Length of hospital Stay						
<21 days	564 (84.4)	628 (94.0)	-	-	-	-
≥21days	104 (15.6)	40 (6.0)	.000	2.90	2.98	4.24

AGA= Appropriate for gestational age, SGA = Small for gestational age, RDS = Respiratory distress syndrome, NEC = necrotizing enterocolitis, EOS = Early onset neonatal sepsis, LOS = Late onset neonatal sepsis

Confidential: For Review Only

DISCUSSION

Comparison of neonatal outcomes of SGA and AGA preterm infants remains controversial, as several investigators have reported conflicting data. In the current study, the rates of RDS and mortality among the two groups were similar. These findings were in line with the findings of Bartal et al.[14] And our findings contradict the reports of Tsai et al. and Sharma et al, who reported an increased risk of RDS and mortality among SGA infants.[13, 26] However, Bartels et al and Tyson et al reported increased risk of death and decreased risk of RDS.[12, 19] The contradicting findings in the literature might be due to the multifactorial nature of outcome of SGA, the cause of SGA and severity of the condition, and duration of intrauterine hypoxia. The GA at birth could modify the physiologic changes and adaptation to extrauterine environment.

The SGA infants were more likely to be delivered by cesarean section (p-value =.000) and their mothers were given prophylactic dexamethasone more often compared to mothers of AGA infants (p-value = .017). Pregnancy induced hypertension was associated with the SGA (p-value =.000) whereas acute obstetric condition such as antepartum hemorrhage, chorioamnionitis, and PROM were more common in mothers of AGA infants. This finding is similar to the report of Boghossian et al.[27]

The rate of early onset neonatal infection was comparable in both groups, however SGA infants had a higher risk of late onset neonatal sepsis. Similarly, SGA infants had an increased risk of NEC and hypoglycemia compared to AGA infants. These findings are consistent with reports of Hasthi et al. from India and Boghossian et al. from USA.[15, 27] These can likely be explained by the severe undernutrition the infants experienced predisposing them to infection, although the

Confidential: For Review Only

mechanisms of how under-nutrition is related to immune suppression is not well understood, there are strong epidemiologic data supporting the link.[28] The increased risk of NEC might be associated with immature gut development that has resulted from intrauterine chronic fetal hypoxia and consequent cardiovascular redistribution of blood flow away from the gastrointestinal tract to vital organs.[29]

SGA infants had a statistically significant increased risk of prolonged hospitalization for more than 21 days, likely related to the severity of the morbidities they had; Sharma et al from USA reported a similar finding in a retrospective study involving 2,530 infants born at ≤36 week.[19] Polycythemia (a venous hematocrit above 65%) can occur as a response to intrauterine hypoxia, the hyper-viscosity of blood associated might result in serious complications.[30] SGA infants are at higher risk of developing polycythemia.[2] Similarly, we found a 3-fold increased risk of polycythemia in SGA compared to AGA infants.

CONCLUSION

The SGA infants in this study had increased risk of hypoglycemia, NEC, late onset neonatal sepsis, polycythemia and prolonged hospitalization. The rates of RDS and neonatal mortality were similar in SGA and AGA infants. Proper antenatal care, timely recognition of high risk pregnancies and right interventions are needed to prevent IUGR and the subsequent SGA related complications. Screening for morbidities associated with SGA, preventive measures and adequate postnatal care could contribute for improvement of neonatal outcomes.

Confidential: For Review Only

ACKNOWLEDGMENTS: We would like to thank Bill and Melinda Gates foundation for funding the SIP study: Our special appreciation goes to all families who participated in the study and all health care workers in the five hospitals who helped us in data collection and completion of the study. And Assistance provided by administrators of neonatal intensive care units and hospital managers was greatly appreciated.

COMPETING INTERESTS: The Authors declare that there is no conflict of interest.

FUNDING: The study was funded by Bill and Melinda Gates foundation

CONTRIBUTORSHIP STATEMENT:

The primary study from which the data was extracted "Causes of Illness and Death of Preterm Infants in Ethiopia (SIP)" was conceptualized and designed by AKN and LMM; data collection was monitored by LMM, BW, EMM, AM and RLG. NWG analyzed the data and drafted the manuscript and RLG, AKN, EMM, AM, BW, MS, OG and LMM contributed in the writing and reviewing the manuscript. All authors have revised the work critically and approved the final manuscript as submitted.

Confidential: For Review Only

What is already known on this topic?

Intrauterine growth restriction is one of the common perinatal complications associated with increased neonatal morbidity and mortality. As an adaptation to early extrauterine life, in utero stress associated with placental insufficiency increases secretion of steroid hormones in the fetus. This results in acceleration of brain and lung maturation, however several studies have reported contradictory findings on the morbidity and mortality patterns of SGA infants.

What this study adds?

Accelerated maturity associated with intrauterine growth restriction expected in SGA preterm infants did not protect them from RDS and mortality. Rather, SGA infants have significantly increased risk of hypoglycemia, necrotizing enterocolitis, polycythemia, late onset neonatal sepsis and prolonged hospitalization. The contradicting findings in the literature might be due to the multifactorial nature of outcome of SGA, which depends on the degree of fetal compromise.

Confidential: For Review Only

REFERENCES

1. De Onis M, Blössner M, Villar JO. Levels and patterns of intrauterine growth retardation in developing countries. *Eur J Clin Nutr* 1998;52:S5.
2. Colella M, Frérot A, Novais ARB, et al. Neonatal and Long-Term Consequences of Fetal Growth Restriction. *Curr Pediatr Rev* 2018;14:212–218.
3. Engineer N, Kumar S. Perinatal variables and neonatal outcomes in severely growth restricted preterm fetuses. *Acta Obstet Gynecol Scand* 2010;89:1174–1181.
4. Garite TJ, Clark R, Thorp JA. Intrauterine growth restriction increases morbidity and mortality among premature neonates. *Am J Obstet Gynecol. Journal* 2004;191:481–487.
5. Physical status: the use and interpretation of anthropometry. Report of a WHO Expert Committee. *World Health Organ Tech Rep Ser* 1995;854:1–452.
6. Ota E, Ganchimeg T, Morisaki N, et al. WHO Multi-Country Survey on Maternal and Newborn Health Research Network. Risk factors and adverse perinatal outcomes among term and preterm infants born small-for-gestational-age: secondary analyses of the WHO Multi-Country Survey on Maternal and Newborn Health. *PLoS One* 2014;9(8).
7. Suhag A, Berghella V. Intrauterine growth restriction (IUGR): etiology and diagnosis. *Current Obstetrics and Gynecology Reports* 2013;2:102–111.
8. Arigliani M, Spinelli AM, Liguoro I, et al. Nutrition and Lung Growth. *Nutrients* 2018;10:919.
9. Amiel-Tison C, Cabrol D, Denver R, et al. Fetal adaptation to stress: Part I: Acceleration of fetal maturation and earlier birth triggered by placental insufficiency in humans. *Early Hum Dev* 2004;78:15–27.

Confidential: For Review Only

10. Nobile S, Marchionni P, Carnielli VP. Neonatal outcome of small for gestational age preterm infants. *Eur J Pediatr* 2017;176:1083–1088.

11. Amiel-Tison C, Pettigrew AG. Adaptive changes in the developing brain during intrauterine stress. *BRAIN DEV JPN* 1991;13:67–76.

12. Bartels DB, Kreienbrock L, Dammann O, et al. Population based study on the outcome of small for gestational age newborns. *Arch Dis Child Fetal Neonatal Ed* 2005;90:F53–9.

13. Tsai LY, Chen YL, Tsou KI, et al. Taiwan Premature Infant Developmental Collaborative Study Group. The impact of small-for-gestational-age on neonatal outcome among very-low-birth-weight infants. *Pediatr Neonatol* 2015;56:10110–7.

14. Fishel Bartal M, Chen HY, Blackwell SC, et al. Neonatal morbidity in late preterm small for gestational age neonates. *J Matern Fetal Neonatal Med* 2019 23:1–6.

15. Hasthi UR, Ashwani N, Kumar CS, et al. Morbidity and Mortality Patterns in Small for Gestational Age versus Appropriate for Gestational Age Preterm Neonates Admitted in Level II Neonatal Intensive Care Unit: A Observational Study. *Int J Sci Study* 2017;4:133–136.

16. Simchen MJ, Beiner ME, Strauss-Liviathan N, et al. Neonatal outcome in growth-restricted versus appropriately grown preterm infants. *Am J Perinatol* 2000;17(04):187–192.

17. Giapros V, Drougia A, Krallis N, et al. Morbidity and mortality patterns in small-for-gestational age infants born preterm. *J Matern Fetal Neonatal Med* 2012;25:153–157.

18. Qiu X, Lodha A, Shah PS, et al. Canadian Neonatal Network. Neonatal outcomes of small for gestational age preterm infants in Canada. *Am. J. Perinatol* 2012 ;29:87–94.

Confidential: For Review Only

19. Sharma P, McKay K, Rosenkrantz TS, et al. Comparisons of mortality and pre-discharge respiratory outcomes in small-for-gestational-age and appropriate-for-gestational-age premature infants. *BMC Pediatr* 2004;4:9.

20. Malhotra A, Allison BJ, Jenkin G, et al. Neonatal morbidities of fetal growth restriction: pathophysiology and impact. *Front Endocrinol* 2019;10:55.

21. Longo S, Bollani L, Decembrino L, et al. Short-term and long-term sequelae in intrauterine growth retardation (IUGR). *J Matern Fetal Neonatal Med* 2013;26:222–225.

22. Katz J, Lee AC, Kozuki N, et al. Mortality risk in preterm and small-for-gestational-age infants in low-income and middle-income countries: a pooled country analysis. *Lancet* 2013;382:417–25.

23. Muhe LM, McClure EM, Mekasha A, et al. A prospective study of causes of illness and death in preterm infants in Ethiopia: the SIP study protocol. *Reprod. Health* 2018 ;15:116.

24. Muhe LM, McClure EM, Nigussie AK, et al. Major causes of death in preterm infants in selected hospitals in Ethiopia (SIP): a prospective, cross-sectional, observational study. *Lancet Glob Health* 2019 ;7:e1130-8.

25. Fenton TR, Kim JH. A systematic review and meta-analysis to revise the Fenton growth chart for preterm infants. *BMC Pediatr* 2013;13:59.

26. Tyson JE, Kennedy K, Broyles S, et al. The small for gestational age infant: accelerated or delayed pulmonary maturation? Increased or decreased survival?. *Pediatrics* 1995;95:534–538.

27. Boghossian NS, Geraci M, Edwards EM, et al. Morbidity and mortality in small for gestational age infants at 22 to 29 weeks' gestation. *Pediatrics* 2018;141:e20172533.

Confidential: For Review Only

28. Jones KD, Berkley JA, Warner JO. Perinatal nutrition and immunity to infection. *Pediatr Allergy Immunol* 2010;21:564–576.

29. Bozzetti V, Tagliabue PE. Enteral feeding of intrauterine growth restriction preterm infants: theoretical risks and practical implications. *Pediatr Med Chir* 2017;39:160

30. Sarkar S, Rosenkrantz TS. Neonatal polycythemia and hyperviscosity. *Semin Fetal Neonatal Med* 2008;13:248–255

Confidential: For Review Only

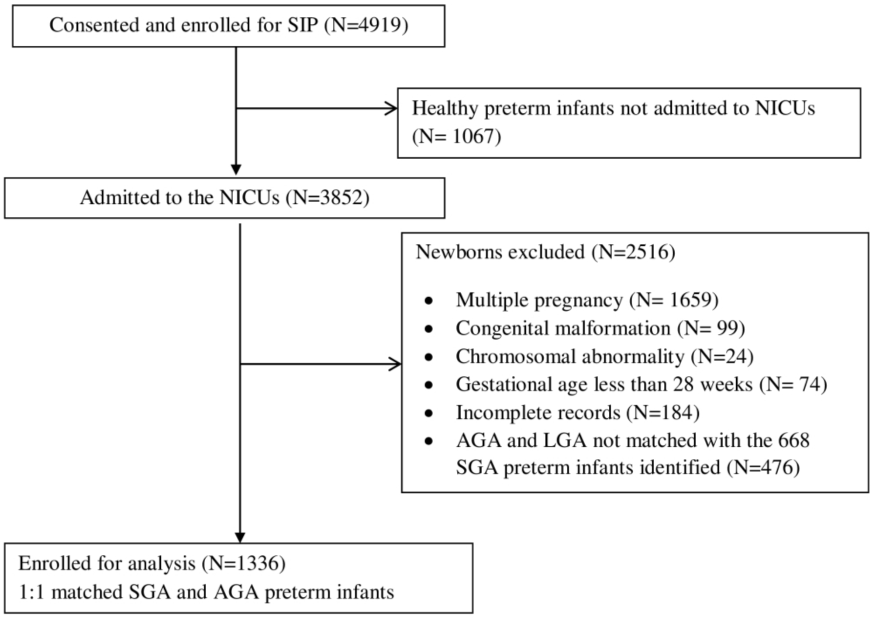
Consented and enrolled for SIP (N=4919)
Healthy preterm infants not admitted to NICUs (N= 1067)
Admitted to the NICUs (N=3852)
Newborns excluded (N=2516)
Multiple pregnancy (N= 1659)
Congenital malformation (N= 99)
Chromosomal abnormality (N=24)
Gestational age less than 28 weeks (N= 74)
Incomplete records (N=184)
AGA and LGA not matched with the 668 SGA preterm infants identified (N=476)
Enrolled for analysis (N=1336)
1:1 matched SGA and AGA preterm infants

BMJ Paediatrics Open

Title: Comparison of neonatal outcomes of small for gestational age and appropriate for gestational age preterm infants born at 28 to 36 weeks of gestation, a multicenter study in Ethiopia

Journal:	*BMJ Paediatrics Open*
Manuscript ID	bmjpo-2020-000740.R1
Article Type:	Original research
Date Submitted by the Author:	25-Jul-2020
Complete List of Authors:	Gidi, Netsanet; Jimma University College of Public Health and Medical Sciences, Pediatric and child health; Ludwig-Maximilians-Universitat Munchen Center for International Health<br>Goldenberg , Robert L. ; Columbia University<br>Nigussie, Assaye K. ; Bill and Melinda Gates Foundation<br>McClure, Elizabeth; RTI International<br>Mekasha, Amha; Addis Ababa University College of Health Sciences<br>Worku, Bogale; Ethiopian Pediatric Society<br>Siebeck, Matthias; Medical Center of the University of Munich (LMU)<br>Genzel-Boroviczeny, Orsolya; Ludwig Maximilian University of Munich , Dr. von Hauner University Children's Hospital<br>Muhe, Lulu M; Addis Ababa University College of Health Sciences
Keywords:	Neonatology, Mortality

SCHOLARONE™
Manuscripts

I, the Submitting Author has the right to grant and does grant on behalf of all authors of the Work (as defined in the below author licence), an exclusive licence and/or a non-exclusive licence for contributions from authors who are: i) UK Crown employees; ii) where BMJ has agreed a CC-BY licence shall apply, and/or iii) in accordance with the terms applicable for US Federal Government officers or employees acting as part of their official duties; on a worldwide, perpetual, irrevocable, royalty-free basis to BMJ Publishing Group Ltd ("BMJ") its licensees and where the relevant Journal is co-owned by BMJ to the co-owners of the Journal, to publish the Work in this journal and any other BMJ products and to exploit all rights, as set out in our licence.

The Submitting Author accepts and understands that any supply made under these terms is made by BMJ to the Submitting Author unless you are acting as an employee on behalf of your employer or a postgraduate student of an affiliated institution which is paying any applicable article publishing charge ("APC") for Open Access articles. Where the Submitting Author wishes to make the Work available on an Open Access basis (and intends to pay the relevant APC), the terms of reuse of such Open Access shall be governed by a Creative Commons licence – details of these licences and which Creative Commons licence will apply to this Work are set out in our licence referred to above.

Other than as permitted in any relevant BMJ Author's Self Archiving Policies, I confirm this Work has not been accepted for publication elsewhere, is not being considered for publication elsewhere and does not duplicate material already published. I confirm all authors consent to publication of this Work and authorise the granting of this licence.

Confidential: For Review Only

Title: Comparison of neonatal outcomes of small for gestational age and appropriate for gestational age preterm infants born at 28 to 36 weeks of gestation, a multicenter study in Ethiopia

Authors: Netsanet Workneh Gidi[a,i], Robert L. Goldenberg[b], Assaye K. Nigussie[c], Elizabeth M. McClure[d], Amha Mekasha[e], Bogale Worku[f], Matthias Siebeck[g], Orsolya Genzel-Boroviczény[h], Lulu M. Muhe[e]

Affiliations

[a] Institute of Health Sciences, Jimma University, Jimma, Ethiopia

[b] Columbia University, New York, NY, USA

[c] Bill and Melinda Gates Foundation, Seattle, WA, USA

[d] RTI International, 3040 Cornwallis Rd., Durham, NC, 27709, USA

[e] College of Health Sciences, Addis Ababa University, Addis Ababa, Ethiopia

[f] Ethiopian Pediatric Society, Addis Ababa, Ethiopia

[g] Medical Center of the University of Munich (LMU), Germany

[h] Dr von Hauner Childrens´Hospital Medical Center of the University of Munich (LMU), Germany

[i] CIH[LMU], Center for International Health, University Hospital, LMU Munich, Germany

Corresponding author: Netsanet Workneh (MD, DTM&H)

College of public health and medical sciences, Jimma University, Jimma, Ethiopia

PhD candidate, at CIHLMU, Munich, Germany

Phone: +251 917762109

Email: konetsanet@gmail.com, or Netsanet.Workneh@lrz.uni-muenchen.de

Word count: 2511

Confidential: For Review Only

ABSTRACT

Purpose: The aim of this study was to assess morbidity and mortality pattern of small for gestational age (SGA) preterm infants in comparison to appropriate for gestational age (AGA) preterm infants of similar gestational age.

Method: We compared neonatal outcomes of 1336, 1:1 matched, singleton SGA and AGA preterm infants based on their gestational age using data from the study "Causes of Illness and Death of Preterm Infants in Ethiopia (SIP)". Data were analyzed using SPSS version 23. Chi square test, odds ratio and 95 % confidence intervals (95%CI) were done, p-value of <0.05 was considered statistically significant.

Result: The majority of the infants 1194 (89%) were moderate to late preterm (32 to 36 weeks of gestation), 763 (57%) were females. Male preterm infants had higher risk of being SGA than female infants, 61.6% versus 41.3%. SGA infants had increased risk of hypoglycemia (OR and 95% CI of 1.6 (1.2-2.0), necrotizing enterocolitis (NEC) 2.3(1.2-4.1), polycythemia 3.0 (1.6-5.4), late onset neonatal sepsis (LOS) 3.6 (1.1-10.9)) and prolonged hospitalization 2.9 (2.0-4.2). The rates of respiratory distress syndrome (RDS), apnea and mortality were similar in the SGA and AGA groups.

Conclusion: Neonatal complications such as hypoglycemia, NEC, LOS, polycythemia and prolonged hospitalization are more common in SGA infants; while rates of RDS and mortality are similar in SGA and AGA groups. Early recognition of SGA status, high index of suspicion and screening for complications associated, and timely intervention to prevent complications need due consideration.

For Review Only

Confidential: For Review

INTRODUCTION

Globally, intrauterine growth restriction (IUGR) occurs in about 24% of newborns per year, and the majority are born in low and middle income countries (LMICs).[1] IUGR is one of the rising public health challenges, because it contributes to increased risk of neonatal morbidity and mortality, and chronic diseases in adulthood.[2-4] Small for gestational age (SGA) is commonly defined as birthweight-for-gestational-age measure below the 10th percentile compared to a gender-specific reference population.[5] IUGR refers to a condition in which the fetal growth is slower than normal, a common cause of SGA; while SGA includes constitutionally small babies.[2] IUGR and SGA can only be distinguished if serial prenatal ultrasound evaluations are done. IUGR occurs due to compromised fetal growth usually related to placental malfunction for various reasons, such as maternal hypertension, diabetes, cardiopulmonary disease, anemia, malnutrition and multiple pregnancies.[6, 7] Congenital malformations and fetal infection may also lead to SGA. IUGR and SGA are commonly used interchangeably and for this paper we will refer to these conditions as SGA.[6]

Conditions, such as multiple pregnancies or pregnancies complicated by maternal hypertension or other placental dysfunction, cause increased secretion of glucocorticoids, other steroid hormones and catecholamines that results in acceleration of brain and lung maturation by as much as 3 to 4 weeks or more when compared to the AGA infants of the same gestational age (GA); this is considered an adaptation to early extrauterine life.[8-10] However, this adaptive

response may fail if the placental dysfunction progresses and the fetus experiences severe anoxia and malnutrition that could result in increased risk of complications and death.[2, 10]

Several studies have reported contradictory findings on the effect IUGR on neonatal respiratory distress syndrome (RDS).[11] The risk of RDS has been reported to be same or lower in SGA infants compared to AGA infants of similar GA,[12-14] but numerous studies have reported increased risk of morbidity and mortality in SGA infants.[3,13,15-19

The reported conflicting findings of neonatal outcomes of SGA infants could be due to the differences in the timing of the onset of placental insufficiency, the severity of growth restriction, and the degree of cardiovascular adaptation [20] Differences in settings of the studies could play a role in terms of early diagnoses of high risk pregnancies and timely intervention, that could abort the progression of the insult and prevent complications.

SGA infants are at higher risk of metabolic and hematological disturbances and those with severe SGA are more likely to die during the neonatal period.[10, 18] Those who survive the neonatal period have a high risk of growth and developmental impairment in childhood, and metabolic, hormonal and cognitive disorders later in adulthood.[7, 21] Reports from high income countries show no significant mortality difference between preterm SGA and AGA infants. However, in LMICs preterm SGA infants have increased risk of mortality. [6] Most of the studies on preterm infants' health and SGA are reported from high income countries and there is a paucity of data from LMICs where the burden is very high.[22] The aim of this study is to assess morbidity and mortality pattern of preterm SGA infants in comparison to AGA infants of similar GA in five neonatal intensive care units (NICUs) in Ethiopia.

METHOD

We analyzed maternal obstetric and clinical data of GA matched SGA and AGA preterm infants admitted to neonatal intensive units (NICUs) from a study on Causes of Illness and Death of Preterm Infants in Ethiopia (SIP). SIP was a prospective descriptive multisite hospital-based study conducted in 5 selected hospitals in Ethiopia. The protocol and the primary result have been published.[23, 24]

After exclusion of multiple births, those with congenital malformations and chromosomal disorders and large for gestational age (LGA) infants, SGA infants were identified and 1:1 match with AGA preterm infants was done randomly. Weight for GA was assessed based on gender and GA specific Fenton growth charts.[25] SGA was defined as a birth weight below the 10th percentile for GA, and AGA birth weight was defined as between the 10th and 90th percentile for GA. The association of SGA with gender, mortality, length of hospital stay, clinical diagnoses such as RDS, necrotizing enterocolitis (NEC), neonatal infections, hypoglycemia, perinatal asphyxia and polycythemia were analyzed. Maternal obstetric variables such as maternal age, marital status, pregnancy induced hypertension, premature rupture of membranes (PROM), antepartum hemorrhage, chorioamnionitis, dexamethasone administration and mode of delivery were assessed for association with birth weight for GA.

GA estimation was based on maternal menstrual history, early fetal ultrasound or New Ballard Score examination. Complications of preterm birth such as RDS and neonatal infections were diagnosed based on clinical findings and investigations including chest x-ray, blood culture and white blood cell count. Death before the 28th day of life was defined as neonatal mortality.

Statistical Analysis was done using the SPSS statistical program. Differences in association of the variables were analyzed with Chi-square tests and a p-value of <0.05 was considered significant. Odds ratios (OR) and 95% confidence intervals (95% CI) were calculated to identify clinical variables associated with SGA.

Ethical approval was obtained from Addis Ababa University College of health Sciences institutional review board (Ethics ID: AAUMF 03-008) before the study was commenced. The parents of the infants were given adequate information and asked for informed consent prior to participation.

Patient and Public Involvement statement

Patients were not involved in the design, recruitment and conduct of this study.

RESULT

A total of 1336 singleton SGA and AGA preterm infants were eligible for the study (Figure1); 763 (57%) were females. The majority of the infants, 1094 (81.9%) were moderate to late preterm (32 to 36 weeks of GA), and 893 (67%) of the infants had a birthweight greater than 1500 grams. The common complications the infants had are shown in table 1.

Table 1. Preterm infants' perinatal data

Variables	No (%)
**Infants sex**	
Male	573 (42.9)
Female	763 (57.1)
**Birthweight in grams**	
<1000	78 (5.8)
1000 to 1499	365 (27.3)
1500 to 1999	538 (40.3)
≥2000	355 (26.6)

Confidential: For Review Only

**Gestational Age in weeks**	
28 to 32	242 (18.1)
33 to 34	562 (42.1)
35 to 36	532 (39.8)
**Common morbidities**	
Neonatal infections	696 (52.1)
Respiratory distress syndrome	514 (38.5)
Hypoglycemia	326 (24.4)
Hyperbilirubinemia	417 (31.2)
Perinatal asphyxia	98 (7.3)
Polycythemia	58 (4.3)
**Distribution of study subjects by hospitals**	
Gondar University hospital	373 (27.9)
Saint Paul Millennium College Hospital	358 (26.8)
Black Lion Hospital	307 (23.0)
Ghandi Memorial Hospital.	158 (11.8)
Jimma University Medical Center	140 (10.5)

The proportion of SGA in male infants was higher than female infants of similar GA, 61.6% versus 41.3%, (p-value <.001). Maternal age and marital status were not associated with SGA; while pregnancy induced hypertension had a statistically significant association with SGA. SGA infants were more likely to be delivered by caesarian section than AGA preterm infants, (p-value<.001). Prophylactic dexamethasone was given more often to the mothers of the preterm infants who were SGA, (p-value = .017). Other obstetric factors studied such as PROM, antepartum hemorrhage, and chorioamnionitis were more common in mothers of AGA preterm infants, (p-value <0.01) (Table 2).

Table 2. Factors associated with birth weight for gestational age.

Variables, No (%)	Total	SGA N=668	AGA N=668	P-value
**Maternal age**				.303
<20 years	236 (17.7)	115 (17.2)	121 (18.1)	
20 to 34 years	980 (73.4)	485 (72.6)	495 (74.1)	

ential: For Review Only

≥35 years	120 (9.0)	88 (13.2)	52 (7.9)	
**Marital Status**				.207
Married	1283 (96.0)	637 (95.4)	647 (96.9)	
Single	53 (4.0)	31(4.6)	21 (3.1)	
**Mode of Delivery**				<.001
Cesarean section	509 (38.1)	283 (42.3)	226 (33.3)	
Vaginal delivery	827 (61.9)	385 (57.3)	442 (66.1)	
**Major obstetric complications**				
Pregnancy induced hypertension	445 (33.3)	290 (43.4)	155 (23.2)	<.001
PROM	191 (14.4)	72 (10.8)	120 (17.9)	<.001
Antepartum hemorrhage	158 (11.8)	63 (9.4)	95 (14.2)	.007
Chorioamnionitis	61 (4.6)	14 (2.1)	47 (7.0)	<.001
Mother received dexamethasone	435 (32.6)	238 (35.6)	197 (29.5)	.017
**Sex of the infant**				<.001
Male	573 (42.9)	353 (61.6)	220 (38.4)	
Female	763 (57.1)	315 (41.3)	448 (58.7)	

AGA= Appropriate for gestational age, SGA = Small for gestational age, PROM= Premature rupture of membranes

The rates of RDS, apnea and mortality were similar among the SGA and AGA groups. While SGA infants had a 1.6 times higher risk of developing hypoglycemia, p-value<.001, OR = 1.58, 95% CI (1.23-2.04). NEC was diagnosed in 5.2% of SGA and 2.4% of AGA infants, p-value =.007, odds ratio = 2.25 95% CI (1.24-4.11). Polycythemia was seen more often in SGA infants, p- value <.001, OR=3.00 95% CI (1.65-5.45). Similar rates of neonatal infections were seen in both groups, while SGA infants had 3.6 times higher risk of developing late onset neonatal sepsis than AGA preterm infants, p-value=.018, OR = 3.55 95%CI (1.16-10.85). SGA infants were more likely to be hospitalized for more than 21 days than AGA preterm infants, p-value<.001, OR=2.90, 95%CI (1.98-4.24) (Table 3).

Confidential: For Review Only

Table 3. Comparison of neonatal outcomes of small for gestational age and appropriate for gestational age preterm infants

Variables	SGA (N=668)	AGA (N=668)	P-value	Odds ratio	95 % CI	
					Lower	Upper
RDS	257 (38.5)	257 (38.5)	1.00	1.00	.80	1.25
Apnea	66 (9.9)	54 (8.0)	.251	1.25	.86	1.82
Hypoglycemia	191 (28.6)	135 (20.2)	<.001	1.58	1.23	2.04
NEC	35 (5.2)	16 (2.4)	.007	2.25	1.24	4.11
Polycythemia	43 (6.4)	15 (2.2)	<.001	3.00	1.65	5.45
EOS	275 (41.2)	271 (40.6)	.824	1.03	.82	1.28
LOS	14 (2.1)	4 (0.6)	.018	3.55	1.16	10.85
Hyperbilirubinemia	196	221	.140	.84	.67	1.06
Perinatal asphyxia	48	50	.834	.96	.63	1.44
Mortality	51 (7.6)	51 (7.6)	1.00	1.00	.67	1.50
Length of hospital Stay						
<21 days	564 (84.4)	628 (94.0)	-	-	-	-
≥21days	104 (15.6)	40 (6.0)	<.001	2.90	2.98	4.24

AGA= Appropriate for gestational age, SGA = Small for gestational age, RDS = Respiratory distress syndrome, NEC = necrotizing enterocolitis, EOS = Early onset neonatal sepsis, LOS = Late onset neonatal sepsis

DISCUSSION

Comparison of neonatal outcomes of SGA and AGA preterm infants remains controversial, as several investigators have reported conflicting results. In the current study, the rates of RDS and mortality among the two groups were similar, unlike the 2 to 4 times, [6] and 16 times increased mortality of SGA preterm infants reported from LMICs. [22] The mortality rate in this study

Confidential: For Review Only

could have been partly modified related to the antenatal dexamethasone the SGA groups had received more than the AGA infants. These findings were in line with the report of Bartal et al among late preterm neonates.[14] And our findings contradict the reports of Tsai et al. and Sharma et al, who reported an increased risk of RDS and mortality among SGA infants.[13, 26] However, Bartels et al and Tyson et al reported increased risk of death and decreased risk of RDS.[12, 19] The contradicting findings in the literature might be due to the multifactorial nature of outcome of SGA, the cause of SGA and severity of the condition, duration of intrauterine hypoxia, and variations in settings of the studies. The GA at birth could modify the physiologic changes and adaptation to extrauterine environment.

The SGA infants were more likely to be delivered by cesarean section (p-value<.001) and their mothers were given prophylactic dexamethasone more often compared to mothers of AGA infants (p-value = .017), this may have improved the overall outcome of the SGA infants. Pregnancy induced hypertension was associated with the SGA (p-value <.001) whereas acute obstetric condition such as antepartum hemorrhage, chorioamnionitis, and PROM were more common in mothers of AGA infants. This finding is similar to the report of Boghossian et al.[27]

The rate of early onset neonatal infection was comparable in both groups, however SGA infants had a higher risk of late onset neonatal sepsis. Similarly, SGA infants had an increased risk of NEC and hypoglycemia compared to AGA infants. These findings are consistent with reports of Hasthi et al. from India and Boghossian et al. from USA.[15, 27] These can likely be explained by the severe undernutrition the infants experienced predisposing them to infection, although the mechanisms of how under-nutrition is related to immune suppression is not well understood, there are strong epidemiologic data supporting the link.[28] The increased risk of NEC might be

Confidential: For Review Only

associated with immature gut development that has resulted from intrauterine chronic fetal hypoxia and consequent cardiovascular redistribution of blood flow away from the gastrointestinal tract to vital organs.[29]

SGA infants had a statistically significant increased risk of prolonged hospitalization for more than 21 days, likely related to the severity of the morbidities they had; Sharma et al from USA reported a similar finding in a retrospective study involving 2,530 infants born at $\leq$36 week.[19] Polycythemia (a venous hematocrit above 65%) can occur as a response to intrauterine hypoxia, the hyper-viscosity of blood associated might result in serious complications.[30] SGA infants are at higher risk of developing polycythemia.[2] Similarly, we found a 3-fold increased risk of polycythemia in SGA compared to AGA infants. The observed complications could be prevented with improvement of neonatal care.

CONCLUSION

The SGA infants in this study had increased risk of hypoglycemia, NEC, late onset neonatal sepsis, polycythemia and prolonged hospitalization. The rates of RDS and neonatal mortality were similar in SGA and AGA infants. Proper antenatal care, timely recognition of high risk pregnancies and right interventions are needed to prevent IUGR and the subsequent SGA related complications. Screening for morbidities associated with SGA, preventive measures and adequate postnatal care could contribute for improvement of neonatal outcomes.

Confidential: For Review Only

ACKNOWLEDGMENTS: We would like to thank Bill and Melinda Gates foundation for funding the SIP study. Our special appreciation goes to all families who participated in the study and all health care workers in the five hospitals who helped us in data collection and completion of the study. And Assistance provided by administrators of neonatal intensive care units and hospital managers was greatly appreciated.

COMPETING INTERESTS: The Authors declare that there is no conflict of interest.

FUNDING: The study was funded by Bill and Melinda Gates foundation, grant number: OPP1136965.

CONTRIBUTORSHIP STATEMENT:

The primary study from which the data was extracted "Causes of Illness and Death of Preterm Infants in Ethiopia (SIP)" was conceptualized and designed by AKN and LMM; data collection was monitored by LMM, BW, EMM, AM and RLG. NWG analyzed the data and drafted the manuscript and RLG, AKN, EMM, AM, BW, MS, OG and LMM contributed in the writing and reviewing the manuscript. All authors have revised the work critically and approved the final manuscript as submitted.

Confidential: For Review Only

What is already known on this topic?

Intrauterine growth restriction is one of the common perinatal complications associated with increased neonatal morbidity and mortality. As an adaptation to early extrauterine life, in utero stress associated with placental insufficiency increases secretion of steroid hormones in the fetus. This results in acceleration of brain and lung maturation, however several studies have reported contradictory findings on the morbidity and mortality patterns of SGA infants.

What this study adds?

Accelerated maturity associated with intrauterine growth restriction expected in SGA preterm infants did not protect them from RDS and mortality. Rather, SGA infants have significantly increased risk of hypoglycemia, necrotizing enterocolitis, polycythemia, late onset neonatal sepsis and prolonged hospitalization.

REFERENCES

1. De Onis M, Blössner M, Villar JO. Levels and patterns of intrauterine growth retardation in developing countries. *Eur J Clin Nutr* 1998;52:S5.
2. Colella M, Frérot A, Novais ARB, et al. Neonatal and Long-Term Consequences of Fetal Growth Restriction. *Curr Pediatr Rev* 2018;14:212–218.
3. Engineer N, Kumar S. Perinatal variables and neonatal outcomes in severely growth restricted preterm fetuses. *Acta Obstet Gynecol Scand* 2010;89:1174–1181.
4. Garite TJ, Clark R, Thorp JA. Intrauterine growth restriction increases morbidity and mortality among premature neonates. *Am J Obstet Gynecol. Journal* 2004;191:481–487.

Confidential: For Review Only

5. Physical status: the use and interpretation of anthropometry. Report of a WHO Expert Committee. *World Health Organ Tech Rep Ser* 1995;854:1–452.

6. Ota E, Ganchimeg T, Morisaki N, et al. WHO Multi-Country Survey on Maternal and Newborn Health Research Network. Risk factors and adverse perinatal outcomes among term and preterm infants born small-for-gestational-age: secondary analyses of the WHO Multi-Country Survey on Maternal and Newborn Health. *PLoS One* 2014;9(8).

7. Suhag A, Berghella V. Intrauterine growth restriction (IUGR): etiology and diagnosis. *Current Obstetrics and Gynecology Reports* 2013;2:102–111.

8. Amiel-Tison C, Cabrol D, Denver R, et al. Fetal adaptation to stress: Part I: Acceleration of fetal maturation and earlier birth triggered by placental insufficiency in humans. *Early Hum Dev* 2004;78:15–27.

9. Nobile S, Marchionni P, Carnielli VP. Neonatal outcome of small for gestational age preterm infants. *Eur J Pediatr* 2017;176:1083–1088.

10. Amiel-Tison C, Pettigrew AG. Adaptive changes in the developing brain during intrauterine stress. *BRAIN DEV JPN* 1991;13:67–76.

11. Arigliani M, Spinelli AM, Liguoro I, et al. Nutrition and Lung Growth. *Nutrients* 2018;10:919.

12. Bartels DB, Kreienbrock L, Dammann O, et al. Population based study on the outcome of small for gestational age newborns. *Arch Dis Child Fetal Neonatal Ed* 2005;90:F53–9.

13. Tsai LY, Chen YL, Tsou KI, et al. Taiwan Premature Infant Developmental Collaborative Study Group. The impact of small-for-gestational-age on neonatal outcome among very-low-birth-weight infants. *Pediatr Neonatol* 2015;56:10110–7.

Confidential: For Review Only

14. Fishel Bartal M, Chen HY, Blackwell SC, et al. Neonatal morbidity in late preterm small for gestational age neonates. *J Matern Fetal Neonatal Med* 2019 23:1–6.

15. Hasthi UR, Ashwani N, Kumar CS, et al. Morbidity and Mortality Patterns in Small for Gestational Age versus Appropriate for Gestational Age Preterm Neonates Admitted in Level II Neonatal Intensive Care Unit: A Observational Study. *Int J Sci Study* 2017;4:133–136.

16. Simchen MJ, Beiner ME, Strauss-Liviathan N, et al. Neonatal outcome in growth-restricted versus appropriately grown preterm infants. *Am J Perinatol* 2000;17(04):187–192.

17. Giapros V, Drougia A, Krallis N, et al. Morbidity and mortality patterns in small-for-gestational age infants born preterm. *J Matern Fetal Neonatal Med* 2012;25:153–157.

18. Qiu X, Lodha A, Shah PS, et al. Canadian Neonatal Network. Neonatal outcomes of small for gestational age preterm infants in Canada. *Am. J. Perinatol* 2012 ;29:87–94.

19. Sharma P, McKay K, Rosenkrantz TS, et al. Comparisons of mortality and pre-discharge respiratory outcomes in small-for-gestational-age and appropriate-for-gestational-age premature infants. *BMC Pediatr* 2004;4:9.

20. Malhotra A, Allison BJ, Jenkin G, et al. Neonatal morbidities of fetal growth restriction: pathophysiology and impact. *Front Endocrinol* 2019;10:55.

21. Longo S, Bollani L, Decembrino L, et al. Short-term and long-term sequelae in intrauterine growth retardation (IUGR). *J Matern Fetal Neonatal Med* 2013;26:222–225.

https://mc.manuscriptcentral.com/bmjpo
Confidential: For Review Only

22. Katz J, Lee AC, Kozuki N, et al. Mortality risk in preterm and small-for-gestational-age infants in low-income and middle-income countries: a pooled country analysis. *Lancet* 2013;382:417–25.

23. Muhe LM, McClure EM, Mekasha A, et al. A prospective study of causes of illness and death in preterm infants in Ethiopia: the SIP study protocol. *Reprod. Health* 2018 ;15:116.

24. Muhe LM, McClure EM, Nigussie AK, et al. Major causes of death in preterm infants in selected hospitals in Ethiopia (SIP): a prospective, cross-sectional, observational study. *Lancet Glob Health* 2019 ;7:e1130-8.

25. Fenton TR, Kim JH. A systematic review and meta-analysis to revise the Fenton growth chart for preterm infants. *BMC Pediatr* 2013;13:59.

26. Tyson JE, Kennedy K, Broyles S, et al. The small for gestational age infant: accelerated or delayed pulmonary maturation? Increased or decreased survival?. *Pediatrics* 1995;95:534–538.

27. Boghossian NS, Geraci M, Edwards EM, et al. Morbidity and mortality in small for gestational age infants at 22 to 29 weeks' gestation. *Pediatrics* 2018;141:e20172533.

28. Jones KD, Berkley JA, Warner JO. Perinatal nutrition and immunity to infection. *Pediatr Allergy Immunol* 2010;21:564–576.

29. Bozzetti V, Tagliabue PE. Enteral feeding of intrauterine growth restriction preterm infants: theoretical risks and practical implications. *Pediatr Med Chir* 2017;39:160

30. Sarkar S, Rosenkrantz TS. Neonatal polycythemia and hyperviscosity. *Semin Fetal Neonatal Med* 2008;13:248–255

Confidential: For Review Only

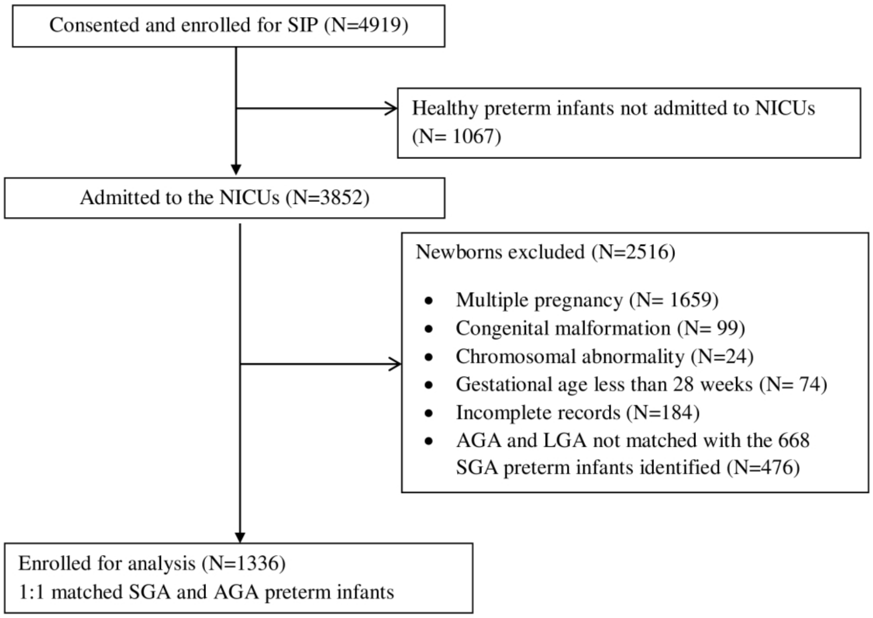
Consented and enrolled for SIP (N=4919)
Healthy preterm infants not admitted to NICUs (N= 1067)
Admitted to the NICUs (N=3852)
Newborns excluded (N=2516)
Multiple pregnancy (N= 1659)
Congenital malformation (N= 99)
Chromosomal abnormality (N=24)
Gestational age less than 28 weeks (N= 74)
Incomplete records (N=184)
AGA and LGA not matched with the 668 SGA preterm infants identified (N=476)
Enrolled for analysis (N=1336)
1:1 matched SGA and AGA preterm infants

BMJ Paediatrics Open

Comparison of neonatal outcomes of small for gestational age and appropriate for gestational age preterm infants born at 28 to 36 weeks of gestation, a multicenter study in Ethiopia

Journal:	*BMJ Paediatrics Open*
Manuscript ID	bmjpo-2020-000740.R2
Article Type:	Original research
Date Submitted by the Author:	08-Aug-2020
Complete List of Authors:	Gidi, Netsanet; Jimma University College of Public Health and Medical Sciences, Pediatric and child health; Ludwig-Maximilians-Universitat Munchen Center for International Health<br>Goldenberg , Robert L. ; Columbia University<br>Nigussie, Assaye K. ; Bill and Melinda Gates Foundation<br>McClure, Elizabeth; RTI International<br>Mekasha, Amha; Addis Ababa University College of Health Sciences<br>Worku, Bogale; Ethiopian Pediatric Society<br>Siebeck, Matthias; Medical Center of the University of Munich (LMU)<br>Genzel-Boroviczeny, Orsolya; Ludwig Maximilian University of Munich , Dr. von Hauner University Children’s Hospital<br>Muhe, Lulu M; Addis Ababa University College of Health Sciences
Keywords:	Neonatology, Mortality

SCHOLARONE™
Manuscripts

I, the Submitting Author has the right to grant and does grant on behalf of all authors of the Work (as defined in the below author licence), an exclusive licence and/or a non-exclusive licence for contributions from authors who are: i) UK Crown employees; ii) where BMJ has agreed a CC-BY licence shall apply, and/or iii) in accordance with the terms applicable for US Federal Government officers or employees acting as part of their official duties; on a worldwide, perpetual, irrevocable, royalty-free basis to BMJ Publishing Group Ltd ("BMJ") its licensees and where the relevant Journal is co-owned by BMJ to the co-owners of the Journal, to publish the Work in this journal and any other BMJ products and to exploit all rights, as set out in our licence.

The Submitting Author accepts and understands that any supply made under these terms is made by BMJ to the Submitting Author unless you are acting as an employee on behalf of your employer or a postgraduate student of an affiliated institution which is paying any applicable article publishing charge ("APC") for Open Access articles. Where the Submitting Author wishes to make the Work available on an Open Access basis (and intends to pay the relevant APC), the terms of reuse of such Open Access shall be governed by a Creative Commons licence – details of these licences and which Creative Commons licence will apply to this Work are set out in our licence referred to above.

Other than as permitted in any relevant BMJ Author's Self Archiving Policies, I confirm this Work has not been accepted for publication elsewhere, is not being considered for publication elsewhere and does not duplicate material already published. I confirm all authors consent to publication of this Work and authorise the granting of this licence.

Confidential: For Review Only

Title: Comparison of neonatal outcomes of small for gestational age and appropriate for gestational age preterm infants born at 28 to 36 weeks of gestation, a multicenter study in Ethiopia

Authors: Netsanet Workneh Gidi[a,i], Robert L. Goldenberg[b], Assaye K. Nigussie[c], Elizabeth M. McClure[d], Amha Mekasha[e], Bogale Worku[f], Matthias Siebeck[g], Orsolya Genzel-Boroviczény[h], Lulu M. Muhe[e]

Affiliations

[a] Institute of Health Sciences, Jimma University, Jimma, Ethiopia

[b] Columbia University, New York, NY, USA

[c] Bill and Melinda Gates Foundation, Seattle, WA, USA

[d] RTI International, 3040 Cornwallis Rd., Durham, NC, 27709, USA

[e] College of Health Sciences, Addis Ababa University, Addis Ababa, Ethiopia

[f] Ethiopian Pediatric Society, Addis Ababa, Ethiopia

[g] Medical Center of the University of Munich (LMU), Germany

[h] Dr von Hauner Childrens´Hospital Medical Center of the University of Munich (LMU), Germany

[i] CIH[LMU], Center for International Health, University Hospital, LMU Munich, Germany

Corresponding author: Netsanet Workneh (MD, DTM&H)

College of public health and medical sciences, Jimma University, Jimma, Ethiopia

PhD candidate, at CIHLMU, Munich, Germany

Phone: +251 917762109

Email: konetsanet@gmail.com, or Netsanet.Workneh@lrz.uni-muenchen.de

Word count: 2506

Confidential: For Review Only

ABSTRACT

Purpose: The aim of this study was to assess morbidity and mortality pattern of small for gestational age (SGA) preterm infants in comparison to appropriate for gestational age (AGA) preterm infants of similar gestational age.

Method: We compared neonatal outcomes of 1336, 1:1 matched, singleton SGA and AGA preterm infants based on their gestational age using data from the study "Causes of Illness and Death of Preterm Infants in Ethiopia (SIP)". Data were analyzed using SPSS version 23. Chi square test, odds ratio and 95 % confidence intervals (95%CI) were done, p-value of <0.05 was considered statistically significant.

Result: The majority of the infants 1194 (89%) were moderate to late preterm (32 to 36 weeks of gestation), 763 (57%) were females. Male preterm infants had higher risk of being SGA than female infants (p-value <0.001).SGA infants had increased risk of hypoglycemia (OR and 95% CI of 1.6 (1.2-2.0), necrotizing enterocolitis (NEC) 2.3(1.2-4.1), polycythemia 3.0 (1.6-5.4), late onset neonatal sepsis (LOS) 3.6 (1.1-10.9)) and prolonged hospitalization 2.9 (2.0-4.2). The rates of respiratory distress syndrome (RDS), apnea and mortality were similar in the SGA and AGA groups.

Conclusion: Neonatal complications such as hypoglycemia, NEC, LOS, polycythemia and prolonged hospitalization are more common in SGA infants; while rates of RDS and mortality are similar in SGA and AGA groups. Early recognition of SGA status, high index of suspicion and screening for complications associated, and timely intervention to prevent complications need due consideration.

For Review Only

INTRODUCTION

Globally, intrauterine growth restriction (IUGR) occurs in about 24% of newborns per year, and the majority are born in low and middle income countries (LMICs).[1] IUGR is one of the rising public health challenges, because it contributes to increased risk of neonatal morbidity and mortality, and chronic diseases in adulthood.[2-4] Small for gestational age (SGA) is commonly defined as birthweight-for-gestational-age measure below the 10th percentile compared to a gender-specific reference population.[5] IUGR refers to a condition in which the fetal growth is slower than normal, a common cause of SGA; while SGA includes constitutionally small babies.[2] IUGR and SGA can only be distinguished if serial prenatal ultrasound evaluations are done. IUGR occurs due to compromised fetal growth usually related to placental malfunction for various reasons, such as maternal hypertension, diabetes, cardiopulmonary disease, anemia, malnutrition and multiple pregnancies.[6, 7] Congenital malformations and fetal infection may also lead to SGA. IUGR and SGA are commonly used interchangeably and for this paper we will refer to these conditions as SGA.[6]

Conditions, such as multiple pregnancies or pregnancies complicated by maternal hypertension or other placental dysfunction, cause increased secretion of glucocorticoids, other steroid hormones and catecholamines that results in acceleration of brain and lung maturation by as much as 3 to 4 weeks or more when compared to the AGA infants of the same gestational age (GA); this is considered an adaptation to early extrauterine life.[8-10] However, this adaptive

Confidential: For Review

response may fail if the placental dysfunction progresses and the fetus experiences severe anoxia and malnutrition that could result in increased risk of complications and death.[2, 10]

Several studies have reported contradictory findings on the effect IUGR on neonatal respiratory distress syndrome (RDS).[11] The risk of RDS has been reported to be same or lower in SGA infants compared to AGA infants of similar GA,[12-14] but numerous studies have reported increased risk of morbidity and mortality in SGA infants.[3,13,15-19

The reported conflicting findings of neonatal outcomes of SGA infants could be due to the differences in the timing of the onset of placental insufficiency, the severity of growth restriction, and the degree of cardiovascular adaptation [20] Differences in settings of the studies could play a role in terms of early diagnoses of high risk pregnancies and timely intervention, that could abort the progression of the insult and prevent complications.

SGA infants are at higher risk of metabolic and hematological disturbances and those with severe SGA are more likely to die during the neonatal period.[10, 18] Those who survive the neonatal period have a high risk of growth and developmental impairment in childhood, and metabolic, hormonal and cognitive disorders later in adulthood.[7, 21] Reports from high income countries show no significant mortality difference between preterm SGA and AGA infants. However, in LMICs preterm SGA infants have increased risk of mortality. [6] Most of the studies on preterm infants' health and SGA are reported from high income countries and there is a paucity of data from LMICs where the burden is very high.[22] The aim of this study is to assess morbidity and mortality pattern of preterm SGA infants in comparison to AGA infants of similar GA in five neonatal intensive care units (NICUs) in Ethiopia.

For Review Only

METHOD

We analyzed maternal obstetric and clinical data of GA matched SGA and AGA preterm infants admitted to neonatal intensive units (NICUs) from a study on Causes of Illness and Death of Preterm Infants in Ethiopia (SIP). SIP was a prospective descriptive multisite hospital-based study conducted in 5 selected hospitals in Ethiopia. The protocol and the primary result have been published.[23, 24]

After exclusion of multiple births, those with congenital malformations and chromosomal disorders and large for gestational age (LGA) infants, SGA infants were identified and 1:1 match with AGA preterm infants was done randomly. Weight for GA was assessed based on gender and GA specific Fenton growth charts.[25] SGA was defined as a birth weight below the 10th percentile for GA, and AGA birth weight was defined as between the 10th and 90th percentile for GA. The association of SGA with gender, mortality, length of hospital stay, clinical diagnoses such as RDS, necrotizing enterocolitis (NEC), neonatal infections, hypoglycemia, perinatal asphyxia and polycythemia were analyzed. Maternal obstetric variables such as maternal age, marital status, pregnancy induced hypertension, premature rupture of membranes (PROM), antepartum hemorrhage, chorioamnionitis, dexamethasone administration and mode of delivery were assessed for association with birth weight for GA.

GA estimation was based on maternal menstrual history, early fetal ultrasound or New Ballard Score examination. Complications of preterm birth such as RDS and neonatal infections were diagnosed based on clinical findings and investigations including chest x-ray, blood culture and white blood cell count. Death before the 28th day of life was defined as neonatal mortality.

Statistical Analysis was done using the SPSS statistical program. Differences in association of the variables were analyzed with Chi-square tests and a p-value of <0.05 was considered significant. Odds ratios (OR) and 95% confidence intervals (95% CI) were calculated to identify clinical variables associated with SGA.

Ethical approval was obtained from Addis Ababa University College of health Sciences institutional review board (Ethics ID: AAUMF 03-008) before the study was commenced. The parents of the infants were given adequate information and asked for informed consent prior to participation.

Patient and Public Involvement statement

Patients were not involved in the design, recruitment and conduct of this study.

RESULT

A total of 1336 singleton SGA and AGA preterm infants were eligible for the study (Figure1); 763 (57%) were females. The majority of the infants, 1094 (81.9%) were moderate to late preterm (32 to 36 weeks of GA), and 893 (67%) of the infants had a birthweight greater than 1500 grams. The common complications the infants had are shown in table 1.

Confidential: For Review Only

Table 1. Preterm infants' perinatal data

Variables	No (%)
**Infants sex**	
Male	573 (42.9)
Female	763 (57.1)
**Birthweight in grams**	
<1000	78 (5.8)
1000 to 1499	365 (27.3)
1500 to 1999	538 (40.3)
≥2000	355 (26.6)
**Gestational Age in weeks**	
28 to 32	242 (18.1)
33 to 34	562 (42.1)
35 to 36	532 (39.8)
**Common morbidities**	
Neonatal infections	696 (52.1)
Respiratory distress syndrome	514 (38.5)
Hypoglycemia	326 (24.4)
Hyperbilirubinemia	417 (31.2)
Perinatal asphyxia	98 (7.3)
Polycythemia	58 (4.3)
**Distribution of study subjects by hospitals**	
Gondar University hospital	373 (27.9)
Saint Paul Millennium College Hospital	358 (26.8)
Black Lion Hospital	307 (23.0)
Ghandi Memorial Hospital.	158 (11.8)
Jimma University Medical Center	140 (10.5)

The male preterm infants had a higher risk of SGA than female infants (p-value <0.001).. Maternal age and marital status were not associated with SGA; while pregnancy induced hypertension had a statistically significant association with SGA. SGA infants were more likely to be delivered by caesarian section than AGA preterm infants, (p-value<.001). Prophylactic dexamethasone was given more often to the mothers of the preterm infants who were SGA, (p-value = .017). Other obstetric factors studied such as PROM, antepartum

Confidential: For Review Only

hemorrhage, and chorioamnionitis were more common in mothers of AGA preterm infants, (p-value <0.01) (Table 2).

Table 2. Factors associated with birth weight for gestational age.

Variables, No (%)	Total	SGA N=668	AGA N=668	P-value
**Maternal age**				.303
<20 years	236 (17.7)	115 (17.2)	121 (18.1)	
20 to 34 years	980 (73.4)	485 (72.6)	495 (74.1)	
≥35 years	120 (9.0)	88 (13.2)	52 (7.9)	
**Marital Status**				.207
Married	1283 (96.0)	637 (95.4)	647 (96.9)	
Single	53 (4.0)	31(4.6)	21 (3.1)	
**Mode of Delivery**				<.001
Cesarean section	509 (38.1)	283 (42.3)	226 (33.3)	
Vaginal delivery	827 (61.9)	385 (57.3)	442 (66.1)	
**Major obstetric complications**				
Pregnancy induced hypertension	445 (33.3)	290 (43.4)	155 (23.2)	<.001
PROM	191 (14.4)	72 (10.8)	120 (17.9)	<.001
Antepartum hemorrhage	158 (11.8)	63 (9.4)	95 (14.2)	.007
Chorioamnionitis	61 (4.6)	14 (2.1)	47 (7.0)	<.001
Mother received dexamethasone	435 (32.6)	238 (35.6)	197 (29.5)	.017
**Sex of the infant**				<.001
Male	573 (42.9)	353 (52.8)	220 (32.9)	
Female	763 (57.1)	315 (47.2)	448 (67.1)	

AGA= Appropriate for gestational age, SGA = Small for gestational age, PROM= Premature rupture of membranes

The rates of RDS, apnea and mortality were similar among the SGA and AGA groups. While SGA infants had a 1.6 times higher risk of developing hypoglycemia, p-value<.001, OR = 1.58, 95% CI (1.23-2.04). NEC was diagnosed in 5.2% of SGA and 2.4% of AGA infants, p-value

Confidential: For Review Only

=.007, odds ratio = 2.25 95% CI (1.24-4.11). Polycythemia was seen more often in SGA infants, p- value <.001, OR=3.00 95% CI (1.65-5.45). Similar rates of neonatal infections were seen in both groups, while SGA infants had 3.6 times higher risk of developing late onset neonatal sepsis than AGA preterm infants, p-value=.018, OR = 3.55 95%CI (1.16-10.85). SGA infants were more likely to be hospitalized for more than 21 days than AGA preterm infants, p-value<.001, OR=2.90, 95%CI (1.98-4.24) (Table 3).

Table 3. Comparison of neonatal outcomes of small for gestational age and appropriate for gestational age preterm infants

Variables	SGA (N=668)	AGA (N=668)	P-value	Odds ratio	95 % CI	
					Lower	Upper
RDS	257 (38.5)	257 (38.5)	1.00	1.00	.80	1.25
Apnea	66 (9.9)	54 (8.0)	.251	1.25	.86	1.82
Hypoglycemia	191 (28.6)	135 (20.2)	<.001	1.58	1.23	2.04
NEC	35 (5.2)	16 (2.4)	.007	2.25	1.24	4.11
Polycythemia	43 (6.4)	15 (2.2)	<.001	3.00	1.65	5.45
EOS	275 (41.2)	271 (40.6)	.824	1.03	.82	1.28
LOS	14 (2.1)	4 (0.6)	.018	3.55	1.16	10.85
Hyperbilirubinemia	196	221	.140	.84	.67	1.06
Perinatal asphyxia	48	50	.834	.96	.63	1.44
Mortality	51 (7.6)	51 (7.6)	1.00	1.00	.67	1.50
Length of hospital Stay						
<21 days	564 (84.4)	628 (94.0)	-	-	-	-
≥21days	104 (15.6)	40 (6.0)	<.001	2.90	2.98	4.24

AGA= Appropriate for gestational age, SGA = Small for gestational age, RDS = Respiratory distress syndrome, NEC = necrotizing enterocolitis, EOS = Early onset neonatal sepsis, LOS = Late onset neonatal sepsis

Confidential: For Review Only

DISCUSSION

Comparison of neonatal outcomes of SGA and AGA preterm infants remains controversial, as several investigators have reported conflicting results. In the current study, the rates of RDS and mortality among the two groups were similar, unlike the 2 to 4 times, [6] and 16 times increased mortality of SGA preterm infants reported from LMICs. [22] The mortality rate in this study could have been partly modified related to the antenatal dexamethasone the SGA groups had received more than the AGA infants. These findings were in line with the report of Bartal et al among late preterm neonates.[14] And our findings contradict the reports of Tsai et al. and Sharma et al, who reported an increased risk of RDS and mortality among SGA infants.[13, 26] However, Bartels et al and Tyson et al reported increased risk of death and decreased risk of RDS.[12, 19] The contradicting findings in the literature might be due to the multifactorial nature of outcome of SGA, the cause of SGA and severity of the condition, duration of intrauterine hypoxia, and variations in settings of the studies. The GA at birth could modify the physiologic changes and adaptation to extrauterine environment.

The SGA infants were more likely to be delivered by cesarean section (p-value<.001) and their mothers were given prophylactic dexamethasone more often compared to mothers of AGA infants (p-value = .017), this may have improved the overall outcome of the SGA infants. Pregnancy induced hypertension was associated with the SGA (p-value <.001) whereas acute obstetric condition such as antepartum hemorrhage, chorioamnionitis, and PROM were more common in mothers of AGA infants. This finding is similar to the report of Boghossian et al.[27]

The rate of early onset neonatal infection was comparable in both groups, however SGA infants had a higher risk of late onset neonatal sepsis. Similarly, SGA infants had an increased risk of

Confidential: For Review Only

NEC and hypoglycemia compared to AGA infants. These findings are consistent with reports of Hasthi et al. from India and Boghossian et al. from USA.[15, 27] These can likely be explained by the severe undernutrition the infants experienced predisposing them to infection, although the mechanisms of how under-nutrition is related to immune suppression is not well understood, there are strong epidemiologic data supporting the link.[28] The increased risk of NEC might be associated with immature gut development that has resulted from intrauterine chronic fetal hypoxia and consequent cardiovascular redistribution of blood flow away from the gastrointestinal tract to vital organs.[29]

SGA infants had a statistically significant increased risk of prolonged hospitalization for more than 21 days, likely related to the severity of the morbidities they had; Sharma et al from USA reported a similar finding in a retrospective study involving 2,530 infants born at ≤36 week.[19] Polycythemia (a venous hematocrit above 65%) can occur as a response to intrauterine hypoxia, the hyper-viscosity of blood associated might result in serious complications.[30] SGA infants are at higher risk of developing polycythemia.[2] Similarly, we found a 3-fold increased risk of polycythemia in SGA compared to AGA infants. The observed complications could be prevented with improvement of neonatal care.

CONCLUSION

The SGA infants in this study had increased risk of hypoglycemia, NEC, late onset neonatal sepsis, polycythemia and prolonged hospitalization. The rates of RDS and neonatal mortality were similar in SGA and AGA infants. Proper antenatal care, timely recognition of high risk pregnancies and right interventions are needed to prevent IUGR and the subsequent SGA related

Confidential: For Review Only

complications. Screening for morbidities associated with SGA, preventive measures and adequate postnatal care could contribute for improvement of neonatal outcomes.

ACKNOWLEDGMENTS: We would like to thank Bill and Melinda Gates foundation for funding the SIP study. Our special appreciation goes to all families who participated in the study and all health care workers in the five hospitals who helped us in data collection and completion of the study. And Assistance provided by administrators of neonatal intensive care units and hospital managers was greatly appreciated.

COMPETING INTERESTS: The Authors declare that there is no conflict of interest.

FUNDING: The study was funded by Bill and Melinda Gates foundation, grant number: OPP1136965.

CONTRIBUTORSHIP STATEMENT:

The primary study from which the data was extracted "Causes of Illness and Death of Preterm Infants in Ethiopia (SIP)" was conceptualized and designed by AKN and LMM; data collection was monitored by LMM, BW, EMM, AM and RLG. NWG analyzed the data and drafted the manuscript and RLG, AKN, EMM, AM, BW, MS, OG and LMM contributed in the writing and reviewing the manuscript. All authors have revised the work critically and approved the final manuscript as submitted.

Confidential: For Review Only

What is already known on this topic?

Intrauterine growth restriction is one of the common perinatal complications associated with increased neonatal morbidity and mortality. As an adaptation to early extrauterine life, in utero stress associated with placental insufficiency increases secretion of steroid hormones in the fetus. This results in acceleration of brain and lung maturation, however several studies have reported contradictory findings on the morbidity and mortality patterns of SGA infants.

What this study adds?

Accelerated maturity associated with intrauterine growth restriction expected in SGA preterm infants did not protect them from RDS and mortality. Rather, SGA infants have significantly increased risk of hypoglycemia, necrotizing enterocolitis, polycythemia, late onset neonatal sepsis and prolonged hospitalization.

REFERENCES

1. De Onis M, Blössner M, Villar JO. Levels and patterns of intrauterine growth retardation in developing countries. *Eur J Clin Nutr* 1998;52:S5.
2. Colella M, Frérot A, Novais ARB, et al. Neonatal and Long-Term Consequences of Fetal Growth Restriction. *Curr Pediatr Rev* 2018;14:212–218.
3. Engineer N, Kumar S. Perinatal variables and neonatal outcomes in severely growth restricted preterm fetuses. *Acta Obstet Gynecol Scand* 2010;89:1174–1181.
4. Garite TJ, Clark R, Thorp JA. Intrauterine growth restriction increases morbidity and mortality among premature neonates. *Am J Obstet Gynecol. Journal* 2004;191:481–487.

Confidential: For Review Only

5. Physical status: the use and interpretation of anthropometry. Report of a WHO Expert Committee. *World Health Organ Tech Rep Ser* 1995;854:1–452.
6. Ota E, Ganchimeg T, Morisaki N, et al. WHO Multi-Country Survey on Maternal and Newborn Health Research Network. Risk factors and adverse perinatal outcomes among term and preterm infants born small-for-gestational-age: secondary analyses of the WHO Multi-Country Survey on Maternal and Newborn Health. *PLoS One* 2014;9(8).
7. Suhag A, Berghella V. Intrauterine growth restriction (IUGR): etiology and diagnosis. *Current Obstetrics and Gynecology Reports* 2013;2:102–111.
8. Amiel-Tison C, Cabrol D, Denver R, et al. Fetal adaptation to stress: Part I: Acceleration of fetal maturation and earlier birth triggered by placental insufficiency in humans. *Early Hum Dev* 2004;78:15–27.
9. Nobile S, Marchionni P, Carnielli VP. Neonatal outcome of small for gestational age preterm infants. *Eur J Pediatr* 2017;176:1083–1088.
10. Amiel-Tison C, Pettigrew AG. Adaptive changes in the developing brain during intrauterine stress. *BRAIN DEV JPN* 1991;13:67–76.
11. Arigliani M, Spinelli AM, Liguoro I, et al. Nutrition and Lung Growth. *Nutrients* 2018;10:919.
12. Bartels DB, Kreienbrock L, Dammann O, et al. Population based study on the outcome of small for gestational age newborns. *Arch Dis Child Fetal Neonatal Ed* 2005;90:F53–9.
13. Tsai LY, Chen YL, Tsou KI, et al. Taiwan Premature Infant Developmental Collaborative Study Group. The impact of small-for-gestational-age on neonatal outcome among very-low-birth-weight infants. *Pediatr Neonatol* 2015;56:10110–7.

Confidential: For Review Only

14. Fishel Bartal M, Chen HY, Blackwell SC, et al. Neonatal morbidity in late preterm small for gestational age neonates. *J Matern Fetal Neonatal Med* 2019 23:1–6.

15. Hasthi UR, Ashwani N, Kumar CS, et al. Morbidity and Mortality Patterns in Small for Gestational Age versus Appropriate for Gestational Age Preterm Neonates Admitted in Level II Neonatal Intensive Care Unit: A Observational Study. *Int J Sci Study* 2017;4:133–136.

16. Simchen MJ, Beiner ME, Strauss-Liviathan N, et al. Neonatal outcome in growth-restricted versus appropriately grown preterm infants. *Am J Perinatol* 2000;17(04):187–192.

17. Giapros V, Drougia A, Krallis N, et al. Morbidity and mortality patterns in small-for-gestational age infants born preterm. *J Matern Fetal Neonatal Med* 2012;25:153–157.

18. Qiu X, Lodha A, Shah PS, et al. Canadian Neonatal Network. Neonatal outcomes of small for gestational age preterm infants in Canada. *Am. J. Perinatol* 2012 ;29:87–94.

19. Sharma P, McKay K, Rosenkrantz TS, et al. Comparisons of mortality and pre-discharge respiratory outcomes in small-for-gestational-age and appropriate-for-gestational-age premature infants. *BMC Pediatr* 2004;4:9.

20. Malhotra A, Allison BJ, Jenkin G, et al. Neonatal morbidities of fetal growth restriction: pathophysiology and impact. *Front Endocrinol* 2019;10:55.

21. Longo S, Bollani L, Decembrino L, et al. Short-term and long-term sequelae in intrauterine growth retardation (IUGR). *J Matern Fetal Neonatal Med* 2013;26:222–225.

Confidential: For Review Only

22. Katz J, Lee AC, Kozuki N, et al. Mortality risk in preterm and small-for-gestational-age infants in low-income and middle-income countries: a pooled country analysis. *Lancet* 2013;382:417–25.

23. Muhe LM, McClure EM, Mekasha A, et al. A prospective study of causes of illness and death in preterm infants in Ethiopia: the SIP study protocol. *Reprod. Health* 2018 ;15:116.

24. Muhe LM, McClure EM, Nigussie AK, et al. Major causes of death in preterm infants in selected hospitals in Ethiopia (SIP): a prospective, cross-sectional, observational study. *Lancet Glob Health* 2019 ;7:e1130-8.

25. Fenton TR, Kim JH. A systematic review and meta-analysis to revise the Fenton growth chart for preterm infants. *BMC Pediatr* 2013;13:59.

26. Tyson JE, Kennedy K, Broyles S, et al. The small for gestational age infant: accelerated or delayed pulmonary maturation? Increased or decreased survival?. *Pediatrics* 1995;95:534–538.

27. Boghossian NS, Geraci M, Edwards EM, et al. Morbidity and mortality in small for gestational age infants at 22 to 29 weeks' gestation. *Pediatrics* 2018;141:e20172533.

28. Jones KD, Berkley JA, Warner JO. Perinatal nutrition and immunity to infection. *Pediatr Allergy Immunol* 2010;21:564–576.

29. Bozzetti V, Tagliabue PE. Enteral feeding of intrauterine growth restriction preterm infants: theoretical risks and practical implications. *Pediatr Med Chir* 2017;39:160

30. Sarkar S, Rosenkrantz TS. Neonatal polycythemia and hyperviscosity. *Semin Fetal Neonatal Med* 2008;13:248–255

Confidential: For Review Only

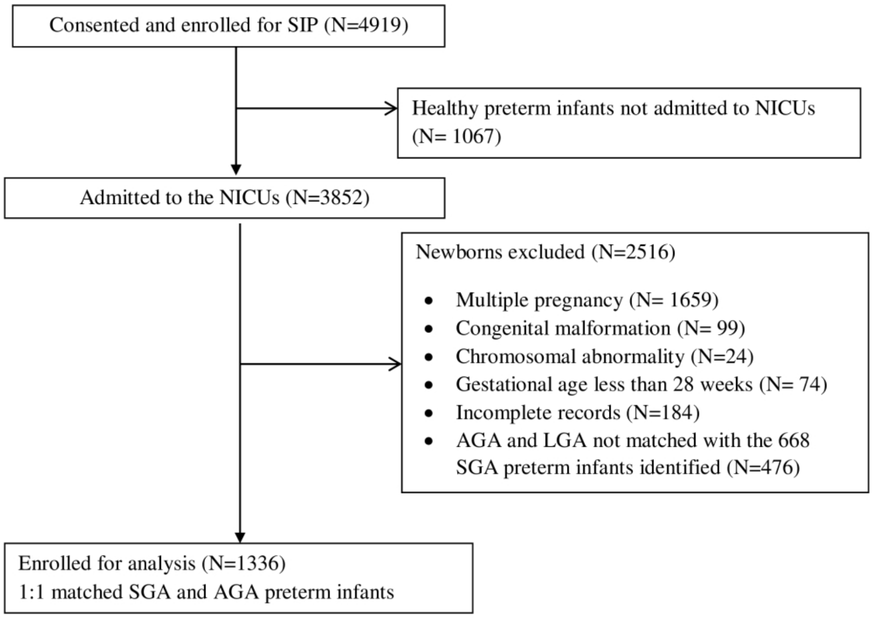
Consented and enrolled for SIP (N=4919)
Healthy preterm infants not admitted to NICUs (N= 1067)
Admitted to the NICUs (N=3852)
Newborns excluded (N=2516)
Multiple pregnancy (N= 1659)
Congenital malformation (N= 99)
Chromosomal abnormality (N=24)
Gestational age less than 28 weeks (N= 74)
Incomplete records (N=184)
AGA and LGA not matched with the 668 SGA preterm infants identified (N=476)
Enrolled for analysis (N=1336)
1:1 matched SGA and AGA preterm infants
